# *Clostridioides difficile* from Fecally Contaminated Environmental Sources: Resistance and Genetic Relatedness from a Molecular Epidemiological Perspective

**DOI:** 10.3390/microorganisms11102497

**Published:** 2023-10-05

**Authors:** Khald Blau, Fabian K. Berger, Alexander Mellmann, Claudia Gallert

**Affiliations:** 1 Department of Microbiology–Biotechnology, Faculty of Technology, University of Applied Sciences Emden/Leer, 26723 Emden, Germany; khald.blau@hs-emden-leer.de; 2 Institute of Medical Microbiology and Hygiene, Saarland University Medical Center, 66421 Homburg, Germany; fabian.berger@uks.eu; 3 German National Reference Center for Clostridioides Difficile, 66421 Homburg, Germany; alexander.mellmann@ukmuenster.de; 4 Institute of Hygiene, University of Münster, 48149 Münster, Germany

**Keywords:** *Clostridioides difficile*, antimicrobial resistance, whole genome sequencing, ribotypes, multi-locus sequence typing, toxin-encoding genes, feces

## Abstract

*Clostridioides difficile* is the most important pathogen causing antimicrobial-associated diarrhea and has recently been recognized as a cause of community-associated *C. difficile* infection (CA-CDI). This study aimed to characterize virulence factors, antimicrobial resistance (AMR), ribotype (RT) distribution and genetic relationship of *C. difficile* isolates from diverse fecally contaminated environmental sources. *C. difficile* isolates were recovered from different environmental samples in Northern Germany. Antimicrobial susceptibility testing was determined by E-test or disk diffusion method. Toxin genes (*tcdA* and *tcdB*), genes coding for binary toxins (*cdtAB*) and ribotyping were determined by PCR. Furthermore, 166 isolates were subjected to whole genome sequencing (WGS) for core genome multi-locus sequence typing (cgMLST) and extraction of AMR and virulence-encoding genes. Eighty-nine percent (148/166) of isolates were toxigenic, and 51% (76/148) were positive for *cdtAB*. Eighteen isolates (11%) were non-toxigenic. Thirty distinct RTs were identified. The most common RTs were RT127, RT126, RT001, RT078, and RT014. MLST identified 32 different sequence types (ST). The dominant STs were ST11, followed by ST2, ST3, and ST109. All isolates were susceptible to vancomycin and metronidazole and displayed a variable rate of resistance to moxifloxacin (14%), clarithromycin (26%) and rifampicin (2%). AMR genes, such as *gyrA/B*, *blaCDD-1/2*, *aph(3′)-llla*-*sat-4*-*ant(6)-la* cassette, *ermB*, *tet*(M), *tet*(40), and *tetA/B*(P), conferring resistance toward fluoroquinolone, beta-lactam, aminoglycoside, macrolide and tetracycline antimicrobials, were found in 166, 137, 29, 32, 21, 72, 17, and 9 isolates, respectively. Eleven “hypervirulent” RT078 strains were detected, and several isolates belonged to RTs (i.e., RT127, RT126, RT023, RT017, RT001, RT014, RT020, and RT106) associated with CA-CDI, indicating possible transmission between humans and environmental sources pointing out to a zoonotic potential.

## 1. Introduction

*Clostridioides difficile* (formerly *Clostridium difficile*) is a Gram-positive, anaerobic, spore-forming, toxin-producing, rod-shaped bacterium, which can cause diarrhea but also more severe disease, such as pseudomembranous colitis and even toxic megacolon [1,2]. CDI usually occurs after antibiotic exposure when the normal gut microbiota is disrupted, giving vegetative and spores of *C. difficile* the ability to thrive. Treatment with antimicrobials, including penicillins, cephalosporins, fluoroquinolones and the macrolide–lincosamide–streptogramin B (MLS_B_) antimicrobials, is considered a high risk factor for CDI development [3,4,5]. 

The pathogenicity of *C. difficile* strains is predominately dependent on the release of two toxins; toxin A (*tcdA*) and toxin B (*tcdB*), which contribute to CDI and the respective genes, are encoded on a 19.6 kb pathogenicity locus (PaLoc) together with the regulatory components, TcdR, TcdC and TcdE [6]. Additionally, binary toxin (CDT) encoded by *cdtAB* is associated with so called “hypervirulent” strains [7]. Besides CDT, these “hypervirulent” strains might harbor mutations in the toxin repressor gene *tcdC*, leading to a higher toxin production [8].

*C. difficile* can be characterized by PCR ribotyping on a molecular level, and several ribotypes (RTs) are of epidemiologic importance. For instance, nosocomial CDI is often associated with “hypervirulent” RT027, which has been frequently found in hospital settings and outbreaks, especially in Europe, North America and to some extent in Asian countries [9,10,11]. Furthermore, other “hypervirulent” RTs, such as RT023, RT078, RT126, RT127, and RT176, are known [12,13,14,15]. Of note, RT078 is more commonly associated with community associated (CA)-CDI. In previous years, the zoonotic potential of *C. difficile* has been under scientific investigation. Several studies have reported that the environment, including animals and food, can be considered as a potential source of CA-CDI [7,16,17,18]. However, up to this date, these reservoirs and *C. difficile* transmission outside the hospital environment are not fully understood.

In recent years, diverse toxigenic *C. difficile* strains were recovered from a broad variety of environmental sources (e.g., food, soil, water, wastewater treatment plants (WWTPs), and animal manure) and from different animal species (e.g., cattle, pig and poultry). This includes common RTs, which are frequently encountered in human disease, such as RT001, RT005, RT014/RT020, RT078, and RT126, [19,20,21,22,23]. The prevalence of RT078, being commonly encountered in pigs, has been one of the frequent RTs in 34 European countries in the year 2008, with 8% [12] with decreasing tendency.

Animal manure and sewage sludge often contains *C. difficile* spores after being treated by digestion or composting in digesters or biogas plants [22,24,25]. Subsequently, the disposal of animal manure and feces, manure-, biogas plant- and thermophilic digester-derived materials or digested sewage sludge as agricultural fertilizers might contribute to environmental contamination with *C. difficile*.

Exemplified for RT078, strains from both humans and animals are genetically related based on subtyping techniques, such as whole genome sequencing (WGS) following by subsequent phylogenetic analysis [13,26,27,28], which demonstrates evidence for zoonotic transmission of *C. difficile* between humans and animals. In particular, WGS provides more-in-depth information about genetic diversity and relatedness resulting in a better understanding of the source and the evolution of *C. difficile* contributing to the current molecular CDI epidemiology [29]. 

Furthermore, the rapid resistance formation in *C. difficile* strains poses a significant threat to global health, driven by the increased use of antimicrobials as a treatment against other intestinal pathogens [3], and is known to promote CDI. Several recent studies have reported the emergence of virulent-resistant bacterial pathogens from a variety of sources, increasing the need for the appropriate use of antimicrobial agents. In *C. difficile*, accessory antimicrobial resistance (AMR) genes are often located on mobile genetic elements (MGEs) (i.e., conjugative and mobilizable transposons, plasmids, and prophages). They can be transferred via horizontal gene transfer (HGT), within toxigenic and non-toxigenic *C. difficile* strains [30] as well as other bacterial species (i.e., *Bacillus subtilis* and *Enterococcus faecalis*) [31,32]. In this study, the strain composition and corresponding phenotypic and genotypic antimicrobial resistance and virulence-associated factors were evaluated giving insight into the molecular epidemiology of *C. difficile* of environmental origin from Northern Germany. In a second step, the genetic relationship between *C. difficile* isolates was determined by using core genome multi-locus sequence typing (cgMLST) based on WGS to show possible epidemiologic intersections.

## 2. Materials and Methods

### 2.1. Isolation and Identification of C. difficile

*C. difficile* isolates used in the present study were recovered from various environmental samples, such as WWTP samples (raw sewage, sewage sludge, activated sewage sludge, and digested sewage sludge), calf feces, cattle feces-contaminated soil, thermophilic digesters for treating biowaste and sewage sludge and digested sewage sludge-amended soils as previously described [22]. Briefly, environmental samples were inoculated in *C. difficile* selective (CD) broth, consisting of proteose peptone 40 g/L, fructose 6.0 g/L, Na_2_HPO_4_ 5.0 g/L, KH_2_PO_4_ 1.0 g/L, MgSO_4_·7H_2_O 0.1 g/L and NaCl 2.0 g/L. Inoculated CD broths were supplemented with (12 mg/L) norfloxacin (Sigma-Aldrich Chemie GmbH, Munich, Germany) and (32 mg/L) moxalactam (Biomol GmbH, Hamburg, Germany) and 0.1% sodium taurocholate (Carl Roth GmbH & Co. KG, Karlsruhe, Germany) for spore germination. All inoculated CD broths were prepared anaerobically in an anaerobic chamber (Coy Laboratory Products, Inc. Los Angeles, CA, USA) and flushed with a gas mixture (80% N_2_ and 20% CO_2_). All inoculated CD broths were incubated at 37 °C for 7–10 days. Each incubated CD broth was then mixed with an equal volume of absolute alcohol (1:1) and incubated at room temperature for 50–60 min. The mixtures were then centrifuged at 4000 rpm for 10 min and the supernatant discarded. The pellet was resuspended in 1× phosphate-buffered saline (PBS) and plated on *Clostridium difficile* agar (CDA, Fisher Scientific GmbH, Schwerte, Germany) supplemented with 7% defibrinated horse blood (Fisher Scientific GmbH, Schwerte, Germany), (12 mg/mL) norfloxacin, (32 mg/mL) moxalactam and 0.1% sodium taurocholate. All plates were incubated anaerobically in anaerobic jars (Schuett-Biotec GmbH, Göttingen, Germany) (80% N_2_, 10% H_2_ and 10% CO_2_) at 37 °C for 2–5 days. Selected colonies were evaluated by morphology and confirmed by the Oxoid *C. difficile* latex agglutination test (Fisher Scientific GmbH, Schwerte, Germany). The final confirmation was made by analyzing the specific housekeeping gene, triose phosphate isomerase (*tpi*), as previously described by Leeme et al. [33].

### 2.2. PCR-Ribotyping and Toxin Genotyping

PCR ribotyping was conducted as described previously [34]. In short, a standardized ESCMID (European Society of Clinical Microbiology and Infectious Diseases) protocol was utilized together with capillary gel electrophoresis. The obtained *C. difficile* isolates were characterized for toxin A (*tcdA*), toxin B (*tcdB*) and binary toxins (CDT, *cdtA B*) by conventional PCR [35], and results were confirmed by analyzing the genome of *C. difficile* strains (see below in Section 2.4).

### 2.3. Antimicrobial Susceptibility Testing 

Antimicrobial susceptibility testing was performed by epsilometry (E-test) and agar disk diffusion as described previously with a McFarland value of 4.0 on Columbia agar (Becton Dickinson, Heidelberg, Germany) [34]. For metronidazole (nitroimidazole), vancomycin (glycopeptide) and moxifloxacin (fluoroquinolone), epsilometry tests were derived from Liofilchem (Roseto degli Abruzzi, Italy) while, for clarithromycin (macrolide) and rifampicin (rifamycin), antibiotic disks originated from Becton Dickinson (Heidelberg, Germany).

### 2.4. Whole Genome Sequencing and Data Analysis

To determine the genetic relationship of the *C. difficile* isolates, 166 isolates were subjected to WGS using the Pacific Biosciences long-read platform Sequel IIe (Pacific Biosciences Inc., Menlo Park, CA, USA) and were subsequently *de novo*-assembled using the SMRT Link software versions 10 and 11 (Pacific Biosciences Inc.) as described recently [36]. For molecular subtyping and to determine the genetic relationship of the different isolates, the cgMLST approach as described elsewhere was applied [37]. Using the Ridom SeqSphere^+^ software version 9 (Ridom GmbH, Münster, Germany), the cgMLST genes were extracted, and a minimum-spanning tree was constructed to display the genotypic clustering. For backwards compatibility, the “classical” MLST Sequence Types (STs) were extracted in accordance to the *C. difficile* MLST database of the PubMLST website (https://pubmlst.org/organisms/clostridioides-difficile/. Accessed 15 November 2022). In addition to the minimum-spanning tree analysis, all single nucleotide polymorphisms (SNPs) were extracted from the cgMLST target genes that were present in all strains investigated, and a phylogenetic tree (neighbor-joining tree) was constructed using the SeqSphere^+^ software. Subsequent graphical representation was done using the iTOL tool version 5 [38]. For further in-depth analysis, the WGS datasets were annotated using the RAST server (the rapid annotation using subsystem technology) version 2.0 (https://rast.nmpdr.org/. Accessed 15 November 2022) [39]. AMR genes were identified by screening contigs with the CARD version 2 (the comprehensive antibiotic resistance databases) using resistance gene identifier (RGI) (https://card.mcmaster.ca/. Accessed 11 April 2023), BacAnt [40], ResFinder 4.1 (https://cge.food.dtu.dk/services/ResFinder/. Accessed 11 April 2023) [41], ARG-ANNOT [42] and Vrprofile2 [43]. The genomes were further analyzed for the presence of known point mutations associated with resistance to fluoroquinolones (e.g., substitution in GyrA and GyrB subunit of the gyrase enzyme) and rifampicin (substitution in RpoB enzyme) using CARD and Snippy v.4.6.0 (https://github.com/tseemann/snippy. Accessed 25 November 2022), respectively.

The toxin genes were identified by using the virulence factors database from BacAnt [40] as well as by annotation provided by the RAST server (https://rast.nmpdr.org/. Accessed 15 November 2022).

All contig sequences generated were submitted to NCBI GenBank under BioProject number (PRJNA1011814).

## 3. Results

The collection of environmental *C. difficile* isolates, which were characterized phenotypically and genotypically in the current study, was obtained from different environmental sources in the Northern region of Germany as described previously [22]. The isolates were characterized for antimicrobial susceptibility patterns, and the genomic characterization was assessed for the RT diversity and the prevalence of virulence-encoding genes and AMR genes. In addition, the genetic relatedness among *C. difficile* isolates was performed using cgMLST based on WGS.

### 3.1. Toxin-Encoding Genes and PCR Ribotypes of C. difficile Strains

In total, 166 *C. difficile* isolates were obtained, 148 (89%) isolates were toxigenic, comprised of *tcdA*^+^/*tcdB*^+^ [72, (49%)], *tcdA*^+^/*tcdB*^+^/*cdtAB*^+^ [76, (51%)] and [18, (11%)] as non-toxigenic isolates (*tcdA*^−^/*tcdB*^−^/*cdtAB*^−^) (Tables S1, S3 and S5). Toxigenic strains could be isolated from almost all environmental samples, (33% in municipal WWTP samples or in feces of calves with 24%).

**Figure 1:**
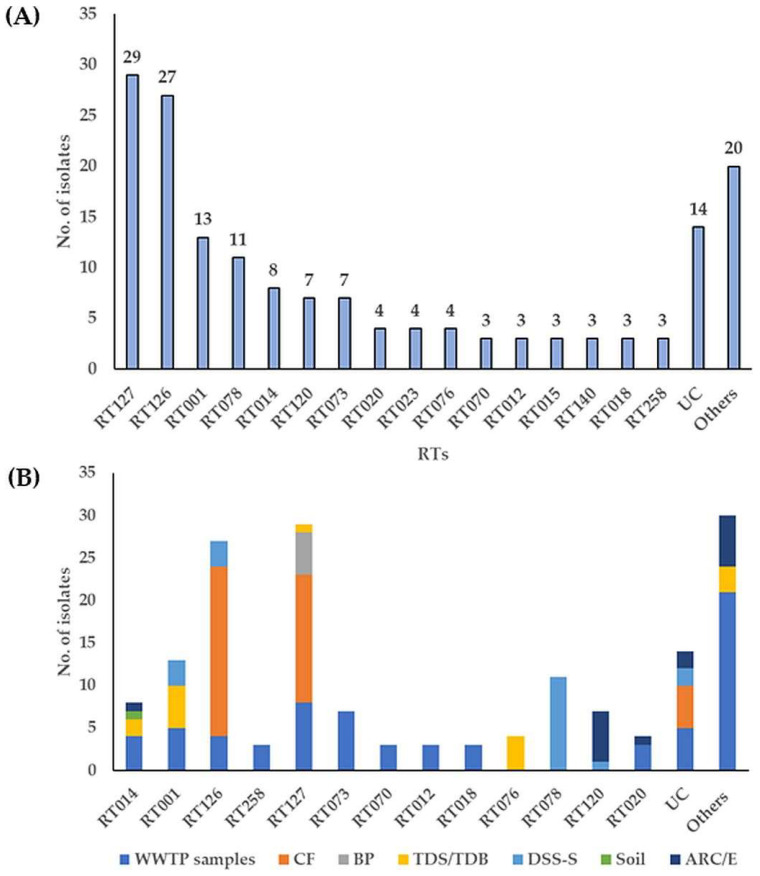
Ribotype (RT) profiling (**A**) and *C. difficile* RTs in environmental samples (**B**). UC: unclassified, CF: calf feces, BP: biogas plant digestate, TDS/TDB: thermophilic digester for treating sewage sludge or biowaste, ARC/E: anaerobic lab-scale bioreactors treating sewage sludge (control and experiment), DSS-S: digested sewage sludge-amended soils. Others indicate RTs with fewer than three assigned strains or samples.

A total of 30 different RT profiles were identified with remaining 14 isolates that could not be classified (UC). Most predominant RTs were RT127 [29, (17%)], RT126 [27, (16%)], RT001 [13, (8%)], RT078 [11, (7%)], and RT014 [8, (5%)], followed by RT120 and RT073 [7, (4%), each] (Figure 1A, Table S2). Among these RTs, municipal WWTP samples, including raw sewage (RS), raw sewage sludge (RSS), digested sewage sludge (DSS), and activated sewage sludge (ASS), showed the greatest diversity (24 different RTs), followed by anaerobic lab scale bioreactors treating sewage sludge supplemented with or without canola lecithin (control/experiment) (ARC/E) (9 RTs), thermophilic digester for treating sewage sludge or biowaste (TDS/TDB) (6 RTs), digested sewage sludge-amended soils (DSS-S) (4 RTs) and calf feces (CF) (2 RTs) (Figure 1B, Table S2). “Hypervirulent” RT027 was absent, however, RT078 was identified only in *C. difficile* isolates recovered from DSS-S [11/20, (55%)] (Figure 1B).

RT126 was found more frequently in isolates from CF [20/40, (50%)], whereas RT127 was predominant in isolates from CF, RSS and biogas plant digestate (BP) [15/40, (38%), 8/23, (35%) and 5/5, (100%), respectively]. RT014 and RT020 were only detected in municipal WWTP samples, TDS/TDB, ARC and cattle feces-contaminated soil, and the prevalence indicates the ubiquitous distribution of this RT (Table S2). Some RTs were rarely identified. This included strains from RS (RT073), DSS (RT258, RT106, and RT103), from TDS (RT076), RS/DSS (RT018), RS/RSS/TDB (RT023), and DSS-S/ARE (RT120) (Figure 1B, Table S2).

**Figure 2:**
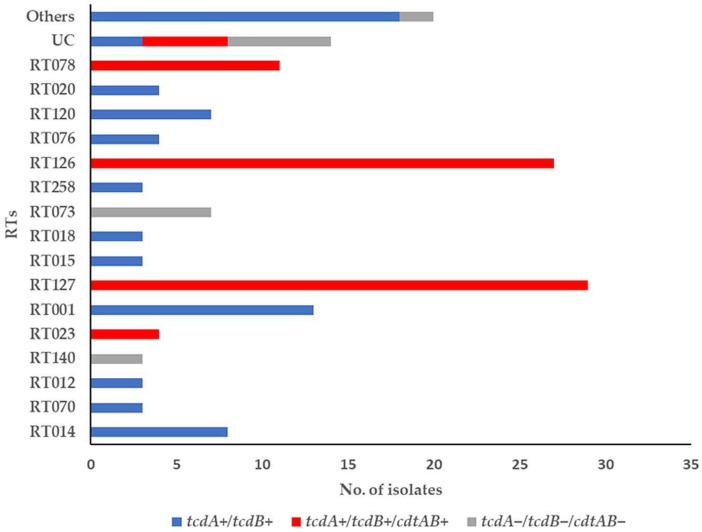
Toxin-encoding genes of *C. difficile* RT strains (*n* = 166) from various environmental samples. UC: unclassified, others indicate RTs with fewer than three assigned strains as follows: 005, 090, 011, 159, 010, 031, 017, 002, 095, 077, 085, 106, 328, and 103.

The toxin-encoding gene profiles of each RT are shown in Figure 2. The most common non-toxigenic strains were RT073 and RT140 [7/18, (39%) and 3/18 (17%), respectively]. The *tcdA*^+^/*tcdB*^+^ was frequently found in *C. difficile* RTs RT001, RT014, RT120, and RT020 [13/72, (18%), 8/72, 11%), 7/72, (10%), and 4/72, (6%), respectively] while the *tcdA*^+^/*tcdB*^+^/*cdtAB*^+^ was identified in *C. difficile* RTs RT126, RT127, RT078, and RT023 [27/76, (36%), 29/76, (38%), 11/76, (14%), and 4/76, (5%), respectively] (Figure 2, Table S3).

### 3.2. Molecular Subtyping, Molecular Epidemiology and Association with RTs and Toxin Genes

**Figure 3:**
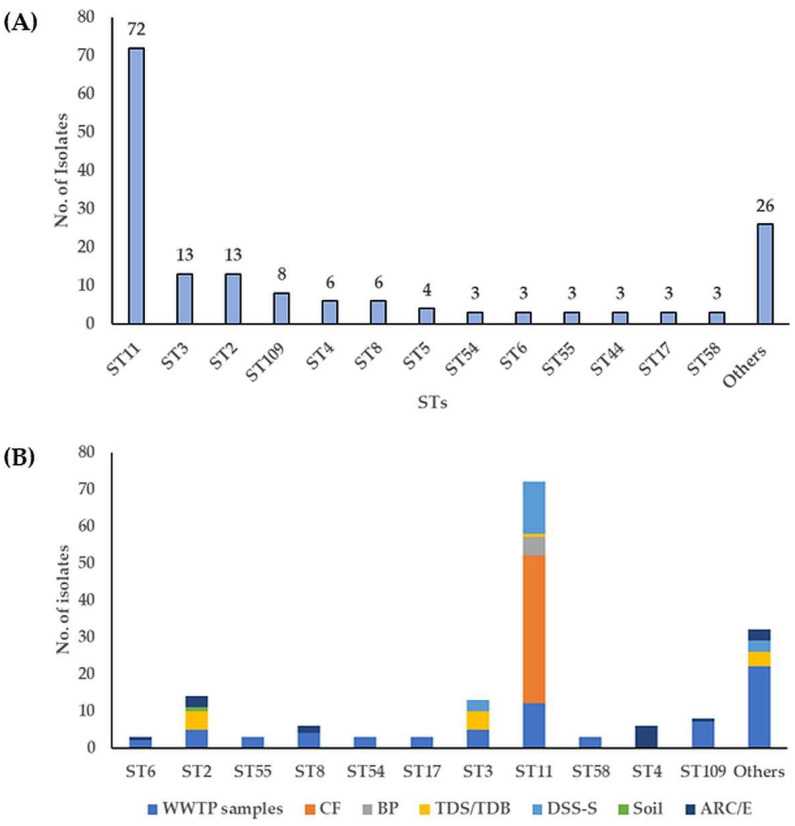
Distribution of *C. difficile* STs (**A**) and in diverse environmental samples (**B**). CF: calf feces, BP: biogas plant digestate, TDS/TDB: thermophilic digester for treating sewage sludge or biowaste, S: soil, ARC/E: anaerobic lab-scale bioreactors treating sewage sludge (control and experiment), DSS-S: digested sewage sludge-amended soils. Others indicate STs with fewer than three assigned strains or samples.

Using MLST, 166 *C. difficile* isolates were classified into 32 different sequence types (STs) (Figure 3A, Table S4). Strains belonging to ST11 were the most common, accounting for [72/ (43%)], followed by those belonging to ST2, ST3, ST109, ST4, and ST8 [14, (8%), 13, (8%), 8, (5%), 7, (4%), and 6, (4%), respectively] (Figure 3A, Table S4). The ST11 was most prevalent in *C. difficile* strains from CF, DSS-S and municipal WWTP samples [40/72, (56%), 14/72 (19%) and 12/72, (17%), respectively] (Figure 3B, Table S4). The ST109 was found only in non-toxigenic *C. difficile* isolates from RS and ARE [7/8, (88%) and 1/8, (13%), respectively] while the ST3 was found in isolates from TDS/TDB, RS, and DSS-S [5/13, (38%), 4/13, (31%), and 3/13, (23%), respectively]. ST4 was identified in strains from ARE [6/7, (86%)], whereas ST2 in strains from municipal WWTP samples (RSS, DSS, and ASS), TDS, ARC, and cattle feces-contaminated soil [5/14, (36%), 5/14, (36%), 3/14, (21%), and 1/14, (7%), respectively]. The ST17 was identified only in municipal WWTP samples (RS and DSS) (Figure 3B, Table S4). The remaining STs were represented by one or two isolates.

**Figure 4:**
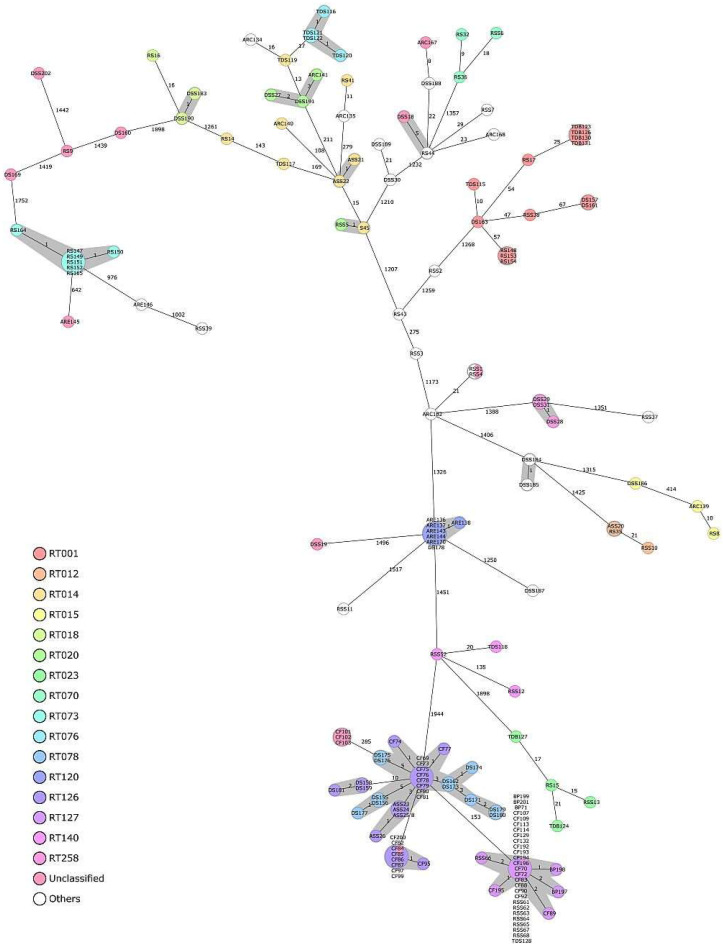
Minimum-spanning tree based on allelic profiles of 166 *C. difficile* isolates. Each circle represents a separate genotype, and distances between two genotypes are based on the allelic profiles of up to 2147 target genes, pairwise ignoring missing targets. The values on the connecting lines indicate the number of allelic differences between the connected isolates. Circle sizes are proportional to the numbers of isolates per genotype (i.e., the allelic profile). Related genotypes (≤6 alleles distance) are shaded in gray, and the isolates are colored according to their RT. RSS: raw sewage sludge, RS: raw sewage, ASS: activated sewage sludge, DSS: digested sewage sludge, CF: calf feces, BP: biogas plant digestate, ARC/E: anaerobic lab-scale bioreactors treating sewage sludge (control and experiment), DS: digested sewage sludge-amended soils, TDS: thermophilic digester for treating sewage sludge, TDB: thermophilic digester for treating biowaste, S: soil.

The results of cgMLST typing and subsequent clustering of the 166 isolates from environmental samples are shown in Figure 4. A minimum-spanning tree was constructed based on the allelic profiles of up to 2147 target genes to display the genotype clustering. Differences detected among the isolates ranged from 0–1944 alleles. In total, cgMLST resulted in discrimination of 98 different genotypes. Of these, 19 genotypes were shared among ≥2 isolates; the remaining 79 genotypes were singletons. Using the cluster threshold of ≤6 cgMLST alleles distance, according to Bletz et al. [37], all isolates formed 20 genotyping clusters consisting of 2 to 32 isolates. The largest cluster consisting of 32 isolates was dominated by isolates of RT127, the second largest cluster (*n* = 25) comprised isolates of RT078 and RT126 (Figure 4, Table S1). Interestingly, genotypes of these two clusters isolates belonged to the same ST11 but differed in cgMLST target genes and their RTs. For instance, RT126 and RT127 isolates differed in 153 cgMLST alleles. Conversely, clustering results indicate that RT126 (10 and 4 isolates from CF and ASS, respectively) is closely related to 11 isolates (RT078) from DSS-S. In addition, among the 32 isolates of the largest cluster, the samples originate from CF, BP, RSS, and TDS. Here, the isolates were distributed based on environmental sources. Also, 14 isolates belonged to the ST2, including different RTs, but the isolate S45 (RT014) showed only one allelic difference from the isolate RSS5 (RT020) whereas the other two isolates (ASS21 and ASS22) remained at 15 allele differences (Figure 4, Table S1).

**Figure 5:**
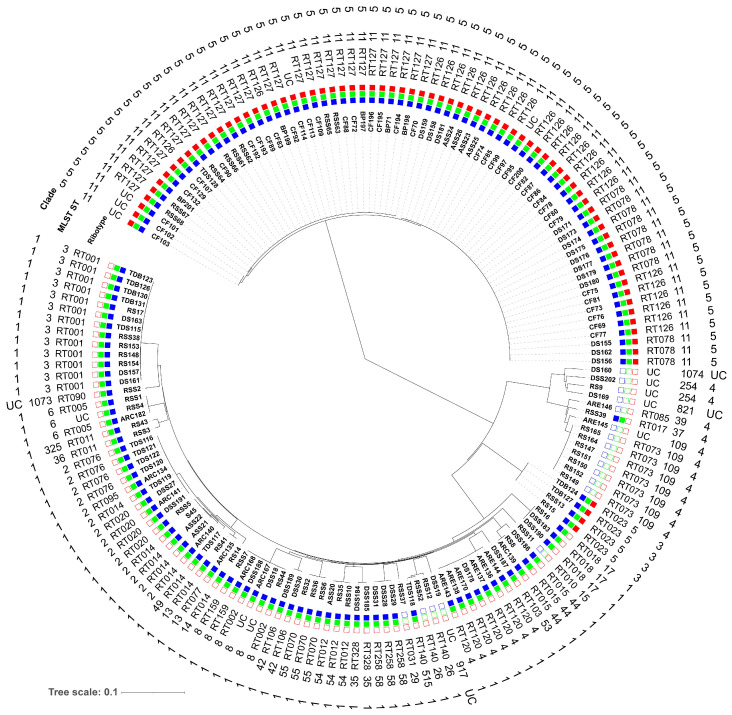
Phylogenetic neighbor-joining tree based on 26,636 SNPs detected in cgMLST genes present in all isolates. In addition, the presence (complete boxes) of toxin genes (*tcdA* [blue], *tcdB* [green], *cdtAB* [red]) and absence (empty boxes), RTs, STs and clades are given. RSS: raw sewage sludge, RS: raw sewage, ASS: activated sewage sludge, DSS: digested sewage sludge, CF: calf feces, BP: biogas plant digestate, ARC/E: anaerobic lab-scale bioreactors treating sewage sludge (control and experiment), DS: digested sewage sludge-amended soils, TDS: thermophilic digester for treating sewage sludge, TDB: thermophilic digester for treating biowaste, S: soil, UC: unclassified.

The SNPs were extracted within the cgMLST dataset to achieve a more in-depth phylogenetic analysis of the 166 *C. difficile* isolates. In total, 26,636 SNPs were extracted and used to construct a phylogenetic neighbor-joining tree (Figure 5). Here, *C. difficile* isolates were grouped by their STs, and four related clusters were displayed. The MLST relationship of the *C. difficile* isolates formed four clades (1, 3, 4, and 5). Clade 1 consists of 21 different STs and clade 4 of four different STs whereas clades 3 and 5 represent one ST each. Clade 1 frequency was higher in municipal WWTP samples. In contrast, clade 5 was more frequent in strains isolated from feces of calves than in municipal WWTP samples (Figure 5, Table S1). Furthermore, some genomes with indistinguishable cgMLST alleles were assigned to multiple RTs, including RT078/RT126 (ST11, clade 5), RT002/RT159 (ST8, clade1), RT077/RT014 (ST13, clade 1), and RT014/RT020/RT076/RT095 (ST2, clade 1). In these cases, several RTs were assigned to different STs and closely related clades (Figure 5, Table 1).

The assignment of *C. difficile* RTs with the STs and MLST clades are also shown in Table 1. The majority of STs correspond to one RT while some correspond to multiple RTs. Four distinct STs were identified in the RT014 collection (STs, 2, 13, 14, and 49; clade 1) while two STs were identified in the RT011 (STs, 36 and 325; clade 1) and in the RT140 (STs, 26 and 515; clade 1). The ST2 has been associated with different RTs, RT020, RT014, ST076, and ST095 in clade 1 (Table 1).

**Figure 6:**
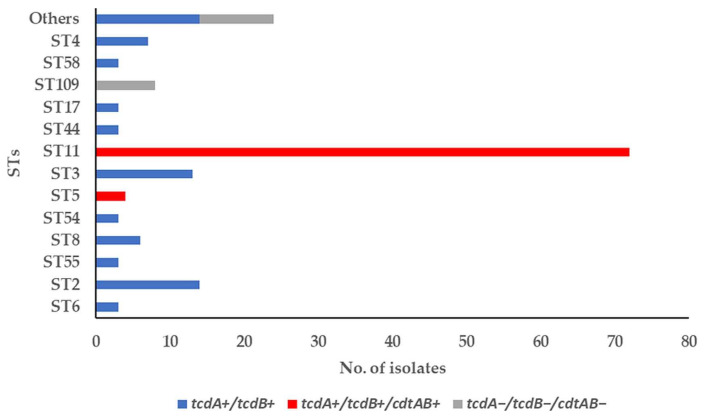
Toxin-encoding genes of *C. difficile* ST strains (*n* = 166) from various environmental samples. Others indicate STs with fewer than three assigned strains as follows: ST1073, ST36, ST15, ST26, ST29, ST37, ST254, ST14, ST325, ST917, ST13, ST35, ST53, ST42, ST515, ST39, ST1074, and ST821.

Interestingly, non-toxigenic strains were found more frequently in clades 4 and 1 while toxigenic strains *tcdA*^+^/*tcdB*^+^ and *tcdA*^+^/*tcdB*^+^/*cdtAB*^+^ were associated with clade 1 and clades 3 and 5, respectively (Figure 5 and Table S1). The toxin-encoding gene profiles of each ST are included in Figure 6. The *tcdA*^+^/*tcdB*^+^/*cdtAB*^+^ and the *tcdA*^+^/*tcdB*^+^ isolates were the dominant profiles [76, (46%) and 72, (43%), respectively]. Of 72 toxigenic strains (*tcdA^+^/tcdB^+^*), 14 (19%), 13 (18%), 7 (10%), and 6 (8%) could be associated with four different STs, ST2, ST3, ST4, and ST8, respectively, all corresponding to clade 1. Whereas 72 out of 76 *tcdA*^+^/*tcdB*^+^/*cdtAB*^+^ strains could be assigned with two different STs, ST11 (clade 5, 95%) and ST5 (clade 3, 5%), respectively (Figure 6, Table S5). Several isolates belonged to STs previously associated with human CA-CDI. The non-toxigenic strains were frequently associated with ST109 [8/18, (44%)] (Figure 6).

### 3.3. Antimicrobial Susceptibility

The antimicrobial susceptibility of 166 *C. difficile* isolates to five tested antibiotics and their corresponding RTs and STs is shown in Table 2 and Table S1. All *C. difficile* strains were susceptible to metronidazole and vancomycin. Overall resistance towards clarithromycin, moxifloxacin and rifampicin was encountered in these strains as follows: 26% (43), 14% (23), and 2% (3), respectively. The most clarithromycin (CLR)-resistant strains were found in CF [18/43, (42%)], municipal WWTP samples [13/43, (30%)], and DSS-S [9/43, (21%)]. In addition, moxifloxacin (MXF)-resistant strains were found in DSS-S and CF [10/23, (43%) and 9/23, (39%), respectively]. The highest number of CLR- and MXF-resistance were observed in *C. difficile* ST11 strains [29/72, (40%) and 17/72, (24%), respectively] (Table 2).

### 3.4. Antimicrobial Resistance (AMR) Genes

**Figure 7:**
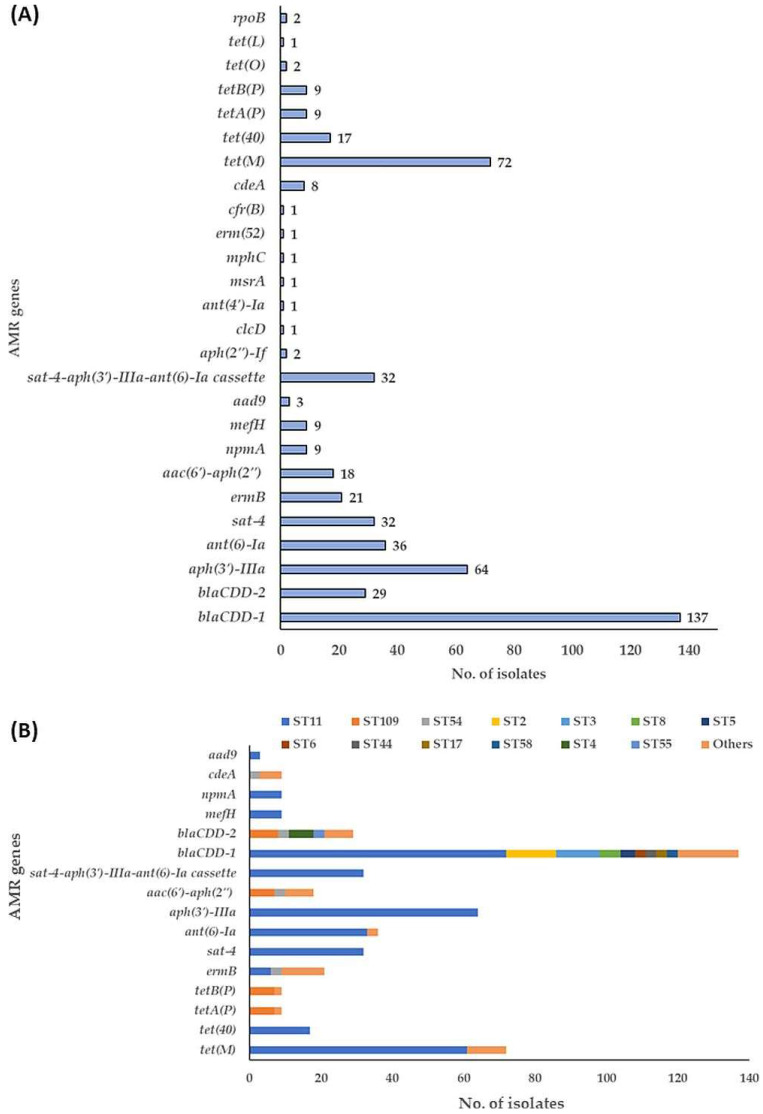
Accessory AMR genes (**A**) and their association with STs (**B**) in environmental *C. difficile* strains (*n* = 166).

All 166 *C. difficile* strains harbored at least four accessory AMR genes (Table S1). The most common accessory AMR genes were *gyrA* and *gyrB,* conferring fluoroquinolone resistance and found in all strains, caused via mutations in the quinolone resistance determining regions (QRDRs) of DNA gyrase subunits A (*gyrA*) and/or B (*gyrB*) (not separately shown in Figure 7). The *blaCDD-1* encoding beta-lactamase could be detected in 137 strains (83%) whereas the *blaCDD-2* gene was found only in 29 strains (17%). The second most abundant resistance gene is *tet*(M) detected in 72 strains (43%) and conferring tetracycline resistance by protecting the ribosomal protection protein. The *aph(3′)-IIIa* gene encoding aminoglycoside resistance was found in 64 strains (39%) whereas *ant(6)-la* gene conferring also aminoglycoside resistance was found in 36 strains (22%). The *sat-4* gene encoding streptothricin resistance was found in 32 strains (19%), and *ermB* encoding a methylase enzyme that protects the 23S rRNA from the binding of the MLS_B_ group antimicrobials was found in 21 strains (13%) (Figure 7A).

Six different tetracycline (*tet*) resistance genes were identified in 85 (51%) out of 166 isolates. Among those *tet* resistance genes, *tet*(M), *tet*(40), *tet*(M)+*tet*(40), *tetA*(P)+*tetB*(P), *tet*(O), and *tet*(L) were found in 72, (85%), 17, (20%), 15, (18%), 9, (11%), 2, (2%), and 1, (1%) isolates, respectively (Figure 7A). The *tet*(M) gene was the most common in isolates recovered from CF and municipal WWTP samples, accounting for [37/72, (51%) and 21/72, (29%), respectively], followed by DSS-S and BP [6/72, (8%) and 5/72, (7%), respectively]. Whereas *tet*(M)+*tet*(40) was more dominant in RT126 isolates from CF and municipal WWTP samples [9/15, (60%), and 4/15, (27%), respectively]. In addition, *tet*(M) was mostly identified in toxigenic *C. difficile* RT126/127 and non-toxigenic *C. difficile* RT140 strains, belonging to ST11 and ST26/515, respectively (Figure 7B). The *tet*(40) gene was found only in RT126 and RT078 (ST11) strains [15/17, (88%) and 2/17, (12%), respectively] isolated from CF, ASS, and DSS-S. Interestingly, *tetA*(P)+*tetB*(P) was identified only in isolates from RS and DSS-S [7/9, (78%) and 2/9, (22%), respectively], and those strains belonged to ST109 (RT073, non-toxigenic strains) and ST3 (RT001, toxigenic strains), respectively (Figure 7B).

Beside isolates carrying more than one tetracycline resistances gene, it was also observed that *C. difficile* isolates harbor one or more genes belonging to an aminoglycoside-streptothricin resistance cassette (*aph(3′)-IIIa-sat-4-ant(6)-la*). Thirty-two strains carried the complete cassette, belonging to ST11 (RT126 and RT078), suggesting that this cluster associated with ST11 while 32 and 4 strains carried only *aph(3′)-IIIa* and *ant(6)-la*, respectively (Figure 7B, Table S1). For aminoglycoside resistance, *aac(6′)-aph(2′’)* gene was identified in [18/166, (11%)] strains, 15 of them were found in isolates from municipal WWTP samples. This gene is frequently associated with two different STs, ST109 and ST54 (Figure 7B).

In addition, another series of genes related to vancomycin resistance, *vanZ1, vanS, vanG* and *vanT* cluster [53/166, (32%)], *vanS, vanG*, and *vanT* cluster [22/166, (13%)] or only *vanZ1* gene [84/166, (51%)] were found in *C. difficile* strains. However, all these isolates, which carried vancomycin resistance clusters, displayed high sensitivity towards vancomycin.

## 4. Discussion

The impact of environmental sources for CDI development is still poorly understood. The presence of toxigenic or non-toxigenic *C. difficile* has been documented in different environmental sources outside healthcare institutions, such as animal feces, manure, soil, food, and municipal WWTPs [17,21,22,24,44], which could be served as potential sources of CA-CDI.

In the present study, a large strain diversity was evident with several strains being of higher epidemiologic importance. In particular, RT014 and RT020 as one of the most often encountered RTs in human disease could be detected together with RT001 which is considered to be a nosocomially associated strain [12]. Furthermore, RT001 and RT014 were one of the most frequently detected in isolates from poultry meat in Germany [19]. RT014 was also detected in soil samples being located next to a dairy farm [45]. RT014 and RT020 were the predominant RT among soil isolates obtained from home gardens in Western Australia [46] and poultry feces [20].

On the other hand, strains that harbor the binary toxin, such as RT126, RT127 and RT078, were present as well. Of note, RT127 was a major clinical strain in Northwestern Taiwan for the years 2009–2015 [14] and was the most numerous RT detected in this study. Moreover, this RT was most frequently found in toxigenic isolates (50.2%) with CDT among obtained RTs from a calf farm in Australia [47].

A similar situation is given for RT126. RT126 was predominately detected in the feces of calves. RT126 has already been described in cattle [21,44] and pigs [44,48]. Furthermore, RT126 has been observed as one of the predominant RTs in a veal calf farm in Belgium [49]. In Spain, RT126 is one of the most common RTs among clinical isolates [48], and RT126 was also identified in clinical isolates in Southern Taiwan [50]. In a study carried out by Primavilla et al. [51] in hospital food in central Italy, RT126 was also the second most frequently detected RT in CDI cases.

Interestingly, RT027 could not be detected in contrast to RT078, being identified with a high prevalence in DSS-S (7%). Of note, RT078, which is commonly associated with CA-CDI, was isolated from 19%, 8%, 35%, and 60% of primary sludge, digested sludge, biosolids, and river sediments, respectively [25], suggesting that RT078 strains might have resistance mechanisms that could enhance its survival during sewage sludge treatment. Furthermore, the RT078 is frequently reported in farm animals, such as cattle [21,44,52,53], poultry [54], and pigs [26,44,55]. Its epidemiologic importance concerning humans might be illustrated in that RT078 was among the five most frequently encountered RTs in Europe [56]. Furthermore, subtyping data conclude a potential for ongoing zoonotic transmission [18,27,44].

RT023 was identified in 2% of isolates being obtained from RS, RSS, and TDB samples. RT023 prevalence, isolated from humans in Europe, was ~3% [12]. Interestingly, RT018 was found in three isolates recovered from municipal WWTP samples (RS and DSS). In the past, RT018 has been associated with a *C. difficile* outbreak in Southern Germany [57]. More importantly, RT018 is considered to be the most predominant RT in Northern Italy with prevalence rates exceeding 40% [58].

Non-toxigenic *C. difficile* strains were identified in particular RT073 (ST109) and RT140 (ST26 and ST515), with prevalence of 4% and 2%, being obtained from RS and (RSS and TDS), respectively. Beside these RTs, one strain each could be assigned to RT010 (ST15) and RT031 (ST29). The presence of non-toxigenic strains is a common finding. Janezic et al. [59] observed that non-toxigenic isolates were commonly found in the environment (30.8%) in comparison to humans (6.5%) and animals (7.7%). Heise et al. [19] observed that several different RTs belonged to non-toxigenic strains, such as RT010, RT205, RT578, RT629, and RT701 obtained from poultry meat in Germany. Interestingly, non-toxigenic ST109 (RT073) was frequently isolated from humans in Japan [60]. 

In summary, concerning molecular epidemiology: RTs being frequently encountered in humans, such as RT001, RT014, and RT020 were present in the collected environmental samples. This might indicate that digested sewage sludge, untreated sewage, raw sewage sludge, biogas plant derived materials and thermophilic digesters treating biowaste or sewage sludge could pose a reservoir of toxigenic *C. difficile* RTs. 

In addition to the classical differentiation of *C. difficile* isolates by ribotyping, the genome sequences were determined as well. This enabled us to further subgroup the isolates. Initially, the grouping was performed based on the cgMLST allelic profiles. This analysis revealed 20 clusters and 47 singletons. Many clusters corroborated with ribotyping results. However, in some instances, cgMLST was unable to group the isolates in accordance with their RTs, e.g., isolates sharing RT078 and RT126 or RT014 and RT020, where the allelic profiles only differed in up to five alleles. This is, however, in agreement with recent studies, which observed clustering of several RTs (e.g., RT078/RT126, RT014/RT020) [61,62]. Here, the current study could demonstrate that the distribution of virulence genes, coding for i.e., the toxins A and B and the binary toxins, is concordant with the phylogenetic branching. This indicates that the different branches, which also represent to some extent the different clades, are stable lineages, and acquisition of the mentioned toxins was an early process during the evolution of these lineages, which goes in line with the clonal population structure [63].

For backwards compatibility, “classical” MLST STs (with seven loci) were also extracted from the genomic data set. Here, 32 distinct STs were determined that showed a good correlation to cgMLST typing results. In contrast, the comparison to ribotyping was not always concordant. For example, isolates of ST11 exhibited different RTs (RT127, RT126 and RT078), which were also separated in most instances using cgMLST. In summary, these results go in line with previous results, where RTs could be correlated with STs only to some extent [63].

*C. difficile* has been known to be resistant to multiple antimicrobials, such as tetracyclines, fluoroquinolones, lincomycin, erythromycin, aminoglycosides, macrolides, and beta-lactam antimicrobials, that are commonly used against bacterial infections in clinical settings [3,5] and continue to be associated with the highest risk for CDI [3]. In the present study, resistance to MXF was frequently detected in ST11 (RT126 and RT078) isolates from the feces of calves and digested sewage sludge-amended soils. Many of RT126 isolates were additionally resistant to CLR, which belongs to the macrolide antibiotic class. These findings are in accordance with what have been reported in calf farms in Italy [21]. Rates of antimicrobial resistance in *C. difficile* differ in diverse geographic regions [4]. In particular, resistance to fluoroquinolones, macrolides, lincosamides, and tetracyclines has been associated with the spread of ST11 sublineages [64]. In addition, *C. difficile* has evolved multiple AMR mechanisms that contribute to the development of AMR in *C. difficile*: (a) harboring of resistance-associated genes in the bacterial chromosome that could be transferred via HGT, including conjugation, transduction or transformation, (b) selection pressure leading to gene mutations, (c) alterations in the antibiotic targets and/or in metabolic pathways in *C. difficile* and (d) biofilm formation [3,65].

In the current study, six different tetracycline resistance genes in 51% of isolates were identified, including *tet*(M), *tet*(40), *tetA*(P), *tetB*(P), *tet*(O), and *tet*(L). The *tet*(M) was the predominant gene of the *tet* class in *C. difficile* strains (43%) and the majority of *C. difficile* RT126 and RT127 isolates were positive for *tet*(M), confirming that tetracycline resistance is widespread among ST11 isolates from a cattle farm. This finding supports the hypothesis of a zoonotic origin of these infections caused by large amounts of tetracyclines used in animal husbandries resulting in a high load released into the agro-ecosystem via organic fertilizers [21,66]. Also, *tet*(M) gene was identified in non-toxigenic *C. difficile* RT140 and RT031 strains. It has been reported that all non-toxigenic *tet*(M)-positive strains from Indonesia and Thailand carried Tn*916* or Tn*5397* transposons [65]. In *C. difficile,* acquired accessory AMR genes are often located on MGEs, and the most common element associated with *tet*(M) mediated tetracycline resistance is Tn*5397* and Tn*916*-like transposons [3,5]. These elements play a crucial role in HGT between distinct toxigenic and non-toxigenic *C. difficile* strains and between *C. difficile* strains and other intestinal pathogens. For instance, Tn*5397* carrying *tet*(M) gene was shown to be transferred from *C. difficile* to *Bacillus subtilis* [31] and *Enterococcus faecalis* [32]. The *tet*(40) gene, which encodes tetracycline efflux, was identified only in RT126 and RT078 isolates which represent 10% from 166 isolates. In a recent study, in 2.1% of 10,330 publicly available *C. difficile* genomes, *tet*(40) gene could be identified [65]. Intriguingly, other *tet* resistance genes, such as *tetA*(P) and *tetB*(P) were found in non-toxigenic RT073 and toxigenic RT001 strains. The *tetA*(P) gene, which mediates active efflux of tetracycline, and *tetB*(P) gene related to ribosomal protection protein family and were first described in anaerobic bacteria, such as *Clostridium perfringens* [67]. Therefore, it is proposed that *tetA*(P) and *tetB*(P) genes are acquired by the conjugative transfer into *C. difficile* from some other pathogenic bacteria. Non-toxigenic strains can act as a reservoir for many AMR genes that could be transferred horizontally to toxigenic strains, as well as to other zoonotic pathogenic bacteria.

Resistance to fluoroquinolones was mediated by the presence of chromosomal mutations in the QRDRs of the *gyrA* and *gyrB* genes. The presence of the mutations in *gyrA* and *gyrB* genes was highly associated with high-risk clones, such as ST11 and ST3, being the most prevalent in the current study. Interestingly, most of obtained amino acid substations patterns in QRDRs of *gyrA* and *gyrB* genes have been previously identified among fluoroquinolone-resistant *C. difficile* strains, belonging to different genotypes, such as RT001, RT018, RT176, and RT046 [68].

Obtained environmental isolates harbored an aminoglycoside-streptothricin resistance cassette (*aph(3′)-IIIa-sat-4-ant*(*6*)*-la*) and were assigned to ST11 (RT126 and RT078), which is similar to the cassette found in *Erysipelothrix rhusiopathiae*, a species commonly found in pig gut [65] and was also detected in *Enterococcus faecium* [69]. The *sat-4* gene was previously detected in *Campylobacter coli* and *Enterococcus faecium* [69,70] and the cassette of resistance genes is found in many bacterial species, indicating the possibility of interspecies transmission. In general, ST11 strains (RT126, RT127, and RT078) show a high proportion of antimicrobial resistance determinates.

For MLS_B_ resistance, the *ermB* gene was identified in 13% of total isolates, which has been associated with CDI outbreaks in Europe [71]. The *ermB* gene is mostly found in the conjugative and mobilizable transposons, Tn*5398*, Tn*6194*, Tn*6218*, and Tn*6215* [3,4].

For vancomycin resistance, multiple *van* gene clusters were identified in obtained *C. difficile* isolates, which were analyzed in this study. However, a complete *van* resistance operon was not detected in these isolates. Several *van* gene clusters, including *vanA*, *vanB*, *vanG*, *vanW*, and *vanZ1*, have been identified in *C. difficile* and associated with high vancomycin minimum inhibitory concentrations (MICs) [72]. The expression of these clusters is controlled by two-component regulatory systems, *vanS* (membrane sensor kinase) and *vanR* (cytoplasmic response regulator) [72,73], suggesting that these clusters were described to be phenotypically silent. Therefore, the presence of *van* resistance clusters in environmental *C. difficile* strains does not always result in their expression in vitro resistance to vancomycin. These strains could be considered susceptible to vancomycin.

For beta-lactam resistance, *blaCDD-1* or *blaCDD-2* genes were detected in all isolates analyzed, which confer resistance against various beta-lactam antibiotics. These enzymes previously identified in *C. difficile* strains allowing to have intrinsic resistance to antimicrobials, such as penicillins and cephalosporins [74], which is highly conserved among those *C. difficile* genomes.

## 5. Conclusions 

This study demonstrated a large genetic overlap between RTs being isolated from environmental samples and humans that may represent a reservoir for CA-CDI. Although RT027 was absent, “hypervirulent” RT078 was found in digested sludge-amended soils, which could possess the ability for zoonotic transmission between humans and environmental sources. Furthermore, a broad variety of AMR genes were predominantly present in the ST11 sublineages. Although resistance to antimicrobials used to treat CDI is rare, this study provides evidence to support the role of AMR in the spread of *C. difficile*. Future studies need to address the question to which extent HGT, e.g., via MGEs (i.e., transposons, prophages, or plasmids), is present—and further triggered by antimicrobial selection pressure—e.g., for the development and emergence of new epidemic strains.

## Figures and Tables

**Table 1:** The ribotypes (RTs) of *C. difficile* linked to STs and MLST clades.

Clade	RT	ST	Clade	RT	ST
Clade 1	RT005 *	ST6	Clade 1	RT031	ST29
	RT090	ST1073		RT001 *	ST3
	RT011 *	ST36, ST325		RT015 *	ST44
	RT020 *	**ST2**		RT014 *	ST14, ST13, **ST2**, ST49
	RT070 *	ST55		RT018 *	ST17
	RT159	ST8		RT002 *	ST8
	RT012 *	ST54		RT258 *	ST58
	RT010	ST15		RT103 *	ST53
	RT140	ST26, ST515	Clade 4	RT085 *	ST39
	RT077 *	ST13		RT017 *	ST37
	RT328 *	ST35		RT073	ST109
	RT106 *	ST42	Clade 5	RT126 *	ST11
	RT076 *	**ST2**		RT127 *	
	RT095	**ST2**		RT078 *	
	RT120	ST4	Clade 3	RT023 *	ST5

(*) Human CA-CDI. STs correspond to more than two RTs marked with bold.

**Table 2:** Antimicrobial resistance profiles of environmental *C. difficile* RT/ST strains (*n* = 166).

RT/ST	No. of Isolates (%)		
	CLR	MXF	RIF
RT126/ST11	24 (89%)	11 (41%)	1 (4%)
RT078/ST11	4 (36%)	5 (45%)	0
RT001/ST3	2 (15%)	2 (15%)	0
RT012/ST54	3 (100%)	0	0
RT140/ST26/ST515	2 (67%)	2 (67%)	0
RT328/ST35	2 (100%)	0	0
RT010/ST15	1 (100%	0	0
RT031/ST29	1 (100%)	0	0
RT017/ST37	0	1 (100%)	1 (100%)
RT106/ST42	1 (50%)	0	0
RT015/ST44	0	1 (33%)	0
RT014/ST2	1 (13%)	0	0
RT085/ST39	1 (100%)	0	1 (100%)
UC/ST11	1 (20%)	1 (20%)	0
Total	43 (26%)	23 (14%)	3 (2%)

MXF: moxifloxacin, CLR: clarithromycin, RIF: rifampicin, UC: unclassified.

## Data Availability

Not applicable.

## Supplementary Materials

The following supporting information can be downloaded at: https://www.mdpi.com/article/10.3390/microorganisms11102497/s1, Table S1: Phenotypic and genotypic characterization of environmental *C. difficile* isolates; Table S2: Distribution of *C. difficile* RTs in diverse environmental samples; Table S3: Toxin-encoding gene profiles of *C. difficile* RT strains (*n* = 166) from various environmental samples; Table S4: Distribution of *C. difficile* STs (*n* = 166) in diverse environmental samples; Table S5: Toxin-encoding gene profiles of *C. difficile* ST strains (*n* = 166) isolated from various environmental samples; Figure S1: Minimum-spanning tree based on allelic profiles of 166 *C. difficile* isolates. Each circle represents a separate genotype, and distances between two genotypes are based on the allelic profiles of up to 2147 target genes, pairwise ignoring missing targets. The values on the connecting lines indicate the number of allelic differences between the connected isolates. Circle sizes are proportional to the numbers of isolates per genotype (i.e., the allelic profile). Related genotypes (≤6 alleles distance) are shaded in gray, and the isolates are colored according to their ST. RSS: raw sewage sludge, RS: raw sewage, ASS: activated sewage sludge, DSS: digested sewage sludge, CF: calf feces, BP: biogas plant digestate, ARC/E: anaerobic lab-scale bioreactors treating sewage sludge (control and experiment), DSS-S: digested sewage sludge-amended soils, TDS: thermophilic digester for treating sewage sludge, TDB: thermophilic digester for treating biowaste, S: soil.

## Abbreviations

CDI	*Clostridioides difficile* infection
HA-CDI	Healthcare associated-CDI
CA-CDI	Community associated-CDI
RT	Ribotype
WGS	Whole-genome sequencing
ST	Sequence type
MLST	Multi-locus sequence typing
cgMLST	Core genome MLST
AMR	Antimicrobial resistance
HGT	Horizontal gene transfer
MGEs	Mobile genetic elements
MXF	Moxifloxacin
CLR	Clarithromycin
RIF	Rifampicin
MLS_B_	Macrolide-lincosamide-streptogramin B
RS	Raw sewage
ASS	Activated sewage sludge
RSS	Raw sewage sludge
DSS	Digested sewage sludge
TDS	Thermophilic digester for sewage sludge
TDB	Thermophilic digester for biowaste
ARC/E	Anaerobic lab scale bioreactors treating sewage sludge (control and experiment)
DSS-S	Digested sewage sludge-amended soils
WWTP	Wastewater treatment plant
CF	Calf feces
BP	Biogas plant digestate
S	Soil
