# Supplementary material for: Clostridioides difficile from Fecally Contaminated Environmental Sources: Resistance and Genetic Relatedness from a Molecular Epidemiological Perspective
Source: Microorganisms. 2023 Oct 5;11(10):2497. doi: 10.3390/microorganisms11102497 (PMC10608975; doi:10.3390/microorganisms11102497)
Supplement: Supplementary file 1 [file microorganisms-11-02497-s001.zip › microorganisms-2620634-supplementary.pdf]

Supplementary Materials

Table S1: Phenotypic and genotypic characterization of environmental *C. difficile* isolates.

Isolate No.	Source	RTs	STs	MLST calde	Toxin genes	Binary toxins	AR profile	AMR genes	Mutations and amino acid substitution
RSS1	RSS	RT005	ST6	1	*tcdA*, *tcdB*			23S rRNA, *blaCDD-1*, *vanZ1*, *vanS*, *vanG*, *vanT*, *gyrA*, *gyrB*	23S rRNA: C665T
RSS2		RT090	ST1073	UC	*tcdA*, *tcdB*			23S rRNA, *blaCDD-1*, *vanZ1*, *vanS*, *vanG*, *vanT*, *cdeA*, *gyrA*, *gyrB*	23S rRNA: C665T
RSS3		RT011	ST36	1	*tcdA*, *tcdB*			23S rRNA, *blaCDD-1*, *vanS*, *vanG*, *vanT*, *cdeA*, *gyrA*, *gyrB*	23S rRNA: C665T
RSS4		UC	ST6	1	*tcdA*, *tcdB*			23S rRNA, *blaCDD-1*, *vanZ1*, *vanS*, *vanG*, *vanT*, *gyrA*, *gyrB*	23S rRNA: C665T
RSS5		RT020	ST2	1	*tcdA*, *tcdB*			23S rRNA, *blaCDD-1*, *vanZ1*, *vanS*, *vanG*, *vanT*, *gyrA*, *gyrB*	23S rRNA: C665T
RSS6		RT070	ST55	1	*tcdA*, *tcdB*			23S rRNA, *blaCDD-2*, *vanZ1*, *vanS*, *vanG*, *vanT*, *gyrA*, *gyrB*	23S rRNA: C665T
RSS7		RT159	ST8	1	*tcdA*, *tcdB*			23S rRNA, *blaCDD-1*, *vanZ1*, *vanS*, *vanG*, *vanT*, *gyrA*, *gyrB*	23S rRNA: C665T
RSS10		RT012	ST54	1	*tcdA*, *tcdB*		CLR	23S rRNA, *blaCDD-2*, *aac(6′)-aph(2′′)*, *ermB*, *cdeA*, *vanZ1*, *vanS*, *vanG*, *vanT*, *gyrA*, *gyrB*	23S rRNA: C665T
RSS11		RT010	ST15	1			CLR	23S rRNA, *blaCDD-2*, *cdeA*, *ermB*, *ant(6)-la*, *vanZ1*, *vanS*, *vanG*, *vanT*, *gyrA*, *gyrB*	23S rRNA: C665T
RSS12		RT140	ST26	1				23S rRNA, *blaCDD-1*, *ermB*, *ant(6)-la*, *tet*(M), *vanZ1*, *vanS*, *vanG*, *vanT*, *gyrA*, *gyrB*	23S rRNA: C665T
RSS13		RT023	ST5	3	*tcdA*, *tcdB*	*cdtAB*		23S rRNA, *blaCDD-1*, *vanZ1*, *gyrA*, *gyrB*	23S rRNA: C665T
RSS37		RT031	ST29	1			CLR	23S rRNA, *blaCDD-1*, *aac(6′)-aph(2′′)*, *ermB*, *tet*(M), *vanZ1*, *vanS*, *vanG*, *vanT*, *gyrA*, *gyrB*	23S rRNA: C665T
RSS38		RT001	ST3	1	*tcdA*, *tcdB*			23S rRNA, *blaCDD-1*, *vanS*, *vanG*, *vanT*, *gyrA*, *gyrB*	23S rRNA: C665T
RSS39		RT017	ST37	4	*tcdA*, *tcdB*		MXF, RIF	23S rRNA, *blaCDD-2*, *vanZ1*, *aac(6′)-aph(2′′)*, , *tet*(M), *cfr*(B), *erm*(52), *clcD*, *gyrA*, *gyrB*, *rpoB*	23S rRNA: C665T<br>*gyrA*: T82I<br>*rpoB*: His502Asn/Arg505Lys
RSS52		RT140	ST26	1			CLR, MXF	23S rRNA, *blaCDD-1*, *vanZ1*, *vanS*, *vanG*, *vanT*, *aph(2′′)-If*, *tet*(M), *tet*(O), *ermB*, *ant(6)-Ia*, *gyrA*, *gyrB*	23S rRNA: C665T
RSS61		RT127	ST11	5	*tcdA*, *tcdB*	*cdtAB*		23S rRNA, *blaCDD-1*, *vanZ1*, *aph(3′)-IIIa*, *tet(M)* , *gyrA*, *gyrB*	23S rRNA: C665T
RSS62		RT127	ST11	5	*tcdA*, *tcdB*	*cdtAB*		23S rRNA, *blaCDD-1*, *vanZ1*, *aph(3′)-IIIa*, *tet(M)* , *gyrA*, *gyrB*	23S rRNA: C665T
RSS63		RT127	ST11	5	*tcdA*, *tcdB*	*cdtAB*		23S rRNA, *blaCDD-1*, *vanZ1*, *aph(3′)-IIIa*, *tet(M)* , *gyrA*, *gyrB*	23S rRNA: C665T
RSS64		RT127	ST11	5	*tcdA*, *tcdB*	*cdtAB*		23S rRNA, *blaCDD-1*, *vanZ1*, *aph(3′)-IIIa*, *tet(M)* , *gyrA*, *gyrB*	23S rRNA: C665T
RSS65		RT127	ST11	5	*tcdA*, *tcdB*	*cdtAB*		23S rRNA, *blaCDD-1*, *vanZ1*, *aph(3′)-IIIa*, *tet(M)* , *gyrA*, *gyrB*	23S rRNA: C665T
RSS66		RT127	ST11	5	*tcdA*, *tcdB*	*cdtAB*		23S rRNA, *blaCDD-1*, *vanZ1*, *aph(3′)-IIIa*, *tet(M)* , *gyrA*, *gyrB*	23S rRNA: C665T
RSS67		RT127	ST11	5	*tcdA*, *tcdB*	*cdtAB*		23S rRNA, *blaCDD-1*, *vanZ1*, *aph(3′)-IIIa*, *tet(M)* , *gyrA*, *gyrB*	23S rRNA: C665T
RSS68		RT127	ST11	5	*tcdA*, *tcdB*	*cdtAB*		23S rRNA, *blaCDD-1*, *vanZ1*, *aph(3′)-IIIa*, *tet(M)* , *gyrA*, *gyrB*	23S rRNA: C665T
RS8	RS	RT015	ST44	1	*tcdA*, *tcdB*			23S rRNA, *blaCDD-1*, *vanZ1*, *vanS*, *vanG*, *vanT*, *gyrA*, *gyrB*	23S rRNA: C665T
RS9		UC	ST254	4				23S rRNA, *blaCDD-2*, *gyrA*, *gyrB*	23S rRNA: C665T
RS14		RT014	ST14	1	*tcdA*, *tcdB*			23S rRNA, *blaCDD-1*, *vanS*, *vanG*, *vanT*, *gyrA*, *gyrB*	23S rRNA: C665T
RS15		RT023	ST5	3	*tcdA*, *tcdB*	*cdtAB*		23S rRNA, *blaCDD-1*, *vanZ1*, *gyrA*, *gyrB*	23S rRNA: C665T
RS16		RT018	ST17	1	*tcdA*, *tcdB*			23S rRNA, *blaCDD-1*, *vanZ1*, *vanS*, *vanG*, *vanT*, *gyrA*, *gyrB*	23S rRNA: C665T
RS17		RT001	ST3	1	*tcdA*, *tcdB*			23S rRNA, *blaCDD-1*, *vanS*, *vanG*, *vanT*, *gyrA*, *gyrB*	23S rRNA: C665T
RS32		RT070	ST55	1	*tcdA*, *tcdB*			23S rRNA, *blaCDD-2*, *vanZ1*, *vanS*, *vanG*, *vanT*, *gyrA*, *gyrB*	23S rRNA: C665T
RS35		RT012	ST54	1	*tcdA*, *tcdB*		CLR	23S rRNA, *blaCDD-2*, *vanZ1*, *vanS*, *vanG*, *vanT*, *cdeA*, *aac(6′)-aph(2′′)*, *ermB*, *tet*(M), *msrA*, *mphC*, *qacR*, *qacA/B*, *ant(4′)-Ia*, *tet*(L) , *gyrA*, *gyrB*	23S rRNA: C665T

Table S1: Continued.

Isolate No.	Source	RTs	STs	MLST calde	Toxin genes	Binary toxins	AR profile	AMR genes	Mutations and amino acid substitution
RS36	RS	RT070	ST55	1	*tcdA*, *tcdB*			23S rRNA, *blaCDD-2*, *vanZ1*, *vanS*, *vanG*, *vanT*, *gyrA*, *gyrB*	23S rRNA: C665T
RS43		RT011	ST325	1	*tcdA*, *tcdB*			23S rRNA, *blaCDD-1*, *vanZ1*, *vanS*, *vanG*, *vanT*, *cdeA*, *gyrA*, *gyrB*	23S rRNA: C665T
RS44		RT002	ST8	1	*tcdA*, *tcdB*			23S rRNA, *blaCDD-1*, *vanZ1*, *vanS*, *vanG*, *vanT*, *gyrA*, *gyrB*	23S rRNA: C665T
RS147		RT073	ST109	4				23S rRNA, *blaCDD-2*, *vanZ1*, *aac(6′)-aph(2′′)*, *ant(6)-la*, *tetB*(P), *tetA*(P) , *gyrA*, *gyrB*	23S rRNA: C665T
RS148		RT001	ST3	1	*tcdA*, *tcdB*			23S rRNA, *blaCDD-1*, *vanZ1*, *vanS*, *vanG*, *vanT*, *gyrA*, *gyrB*	23S rRNA: C665T
RS149		RT073	ST109	4				23S rRNA, *blaCDD-2*, *vanZ1*, *aac(6′)-aph(2′′)*, *ant(6)-la*, *tetB*(P), *tetA*(P) , *gyrA*, *gyrB*	23S rRNA: C665T
RS150		RT073	ST109	4				23S rRNA, *blaCDD-2*, *vanZ1*, *aac(6′)-aph(2′′)*, *ant(6)-la*, *tetB*(P), *tetA*(P) , *gyrA*, *gyrB*	23S rRNA: C665T
RS151		RT073	ST109	4				23S rRNA, *blaCDD-2*, *vanZ1*, *aac(6′)-aph(2′′)*, *ant(6)-la*, *tetB*(P), *tetA*(P) , *gyrA*, *gyrB*	23S rRNA: C665T
RS152		RT073	ST109	4				23S rRNA, *blaCDD-2*, *vanZ1*, *aac(6′)-aph(2′′)*, *ant(6)-la*, *tetB*(P), *tetA*(P) , *gyrA*, *gyrB*	23S rRNA: C665T
RS153		RT001	ST3	1	*tcdA*, *tcdB*			23S rRNA, *blaCDD-1*, *vanZ1*, *vanS*, *vanG*, *vanT*, *gyrA*, *gyrB*	23S rRNA: C665T
RS154		RT001	ST3	1	*tcdA*, *tcdB*			23S rRNA, *blaCDD-1*, *vanZ1*, *vanS*, *vanG*, *vanT*, *gyrA*, *gyrB*	23S rRNA: C665T
RS164		RT073	ST109	4				23S rRNA, *blaCDD-2*, *aac(6′)-aph(2′′)*, *ant(6)-la*, *tetB*(P), *tetA*(P) , *gyrA*, *gyrB*	23S rRNA: C665T
RS165		RT073	ST109	4				23S rRNA, *blaCDD-2*, *vanZ1*, *aac(6′)-aph(2′′)*, *ant(6)-la*, *tetB*(P), *tetA*(P) , *gyrA*, *gyrB*	23S rRNA: C665T
DSS18	DSS	UC	ST8	1	*tcdA*, *tcdB*			23S rRNA, *blaCDD-1*, *vanZ1*, *vanS*, *vanG*, *vanT*, *gyrA*, *gyrB*	23S rRNA: C665T
DSS19		UC	ST917	UC				23S rRNA, *blaCDD-2*, *vanZ1*, *vanS*, *vanG*, *vanT*, *gyrA*, *gyrB*	23S rRNA: C665T
DSS26		RT126	ST11	5	*tcdA*, *tcdB*	*cdtAB*	CLR	23S rRNA, *blaCDD-1*, *vanZ1*, *aph(3′)-IIIa*, *ant(6)-Ia*, *tet*(40), *tet*(M), *sat-4*, *gyrA*, *gyrB*	23S rRNA: C665T
DSS27		RT020	ST2	1	*tcdA*, *tcdB*			23S rRNA, *blaCDD-1*, *vanS*, *vanG*, *vanT*, *gyrA*, *gyrB*	23S rRNA: C665T
DSS28		RT258	ST58	1	*tcdA*, *tcdB*			23S rRNA, *blaCDD-1*, *vanS*, *vanG*, *vanT*, *gyrA*, *gyrB*	23S rRNA: C665T
DSS29		RT258	ST58	1	*tcdA*, *tcdB*			23S rRNA, *blaCDD-1*, *vanS*, *vanG*, *vanT*, *gyrA*, *gyrB*	23S rRNA: C665T
DSS30		RT106	ST42	1	*tcdA*, *tcdB*			23S rRNA, *blaCDD-1*, *vanS*, *vanG*, *vanT*, *gyrA*, *gyrB*	23S rRNA: C665T
DSS31		RT258	ST58	1	*tcdA*, *tcdB*			23S rRNA, *blaCDD-1*, *vanS*, *vanG*, *vanT*, *gyrA*, *gyrB*	23S rRNA: C665T
DSS41		RT014	ST13	1	*tcdA*, *tcdB*			23S rRNA, *blaCDD-1*, *vanZ1*, *vanS*, *vanG*, *vanT*, *gyrA*, *gyrB*	23S rRNA: C665T
DSS183		RT018	ST17	1	*tcdA*, *tcdB*			23S rRNA, *blaCDD-1*, *vanZ1*, *vanS*, *vanG*, *vanT*, *gyrA*, *gyrB*	23S rRNA: C665T
DSS184		RT328	ST35	1	*tcdA*, *tcdB*		CLR	23S rRNA, *blaCDD-1*, *vanS*, *vanG*, *vanT*, *tet(M)*, *ermB*, *aac(6′)-aph(2′′)*, *gyrA*, *gyrB*	23S rRNA: C665T
DSS185		RT328	ST35	1	*tcdA*, *tcdB*		CLR	23S rRNA, *blaCDD-1*, *vanS*, *vanG*, *vanT*, *tet(M)*, *ermB*, *aac(6′)-aph(2′′)* , *gyrA*, *gyrB*	23S rRNA: C665T
DSS186		RT015	ST44	1	*tcdA*, *tcdB*			23S rRNA, *blaCDD-1*, *vanZ1*, *vanS*, *vanG*, *vanT*, *gyrA*, *gyrB*	23S rRNA: C665T
DSS187		RT103	ST53	1	*tcdA*, *tcdB*			23S rRNA, *blaCDD-1*, *vanZ1*, *vanS*, *vanG*, *vanT*, *gyrA*, *gyrB*	23S rRNA: C665T
DSS188		RT002	ST8	1	*tcdA*, *tcdB*			23S rRNA, *blaCDD-1*, *vanZ1*, *vanS*, *vanG*, *vanT*, *gyrA*, *gyrB*	23S rRNA: C665T
DSS189		RT106	ST42	1	*tcdA*, *tcdB*		CLR	23S rRNA, *blaCDD-1*, *vanZ1*, *vanS*, *vanG*, *vanT*, *tet(M)*, *ermB*, *aac(6′)-aph(2′′)* , *gyrA*, *gyrB*	23S rRNA: C665T
DSS190		RT018	ST17	1	*tcdA*, *tcdB*			23S rRNA, *blaCDD-1*, *vanZ1*, *vanS*, *vanG*, *vanT*, *gyrA*, *gyrB*	23S rRNA: C665T
DSS191		RT020	ST2	1	*tcdA*, *tcdB*			23S rRNA, *blaCDD-1*, *vanS*, *vanG*, *vanT*, *gyrA*, *gyrB*	23S rRNA: C665T
DSS202		UC	ST254	4				23S rRNA, *blaCDD-2*, *vanZ1*, *gyrA*, *gyrB*	23S rRNA: C665T
ASS20	ASS	RT012	ST54	1	*tcdA*, *tcdB*		CLR	23S rRNA, *blaCDD-2*, *aac(6′)-aph(2′′)*, *ermB*, *tet*(M), *cdeA*, *vanZ1*, *vanS*, *vanG*, *vanT*, *gyrA*, *gyrB*	23S rRNA: C665T
ASS21		RT014	ST2	1	*tcdA*, *tcdB*			23S rRNA, *blaCDD-1*, *vanZ1*, *vanS*, *vanG*, *vanT*, *gyrA*, *gyrB*	23S rRNA: C665T
ASS22		RT014	ST2	1	*tcdA*, *tcdB*			23S rRNA, *blaCDD-1*, *vanZ1*, *vanS*, *vanG*, *vanT*, *gyrA*, *gyrB*	23S rRNA: C665T

Table S1: Continued.

Isolate No.	Source	RTs	STs	MLST calde	Toxin genes	Binary toxins	AR profile	AMR genes	Mutations and amino acid substitution
ASS23	ASS	RT126	ST11	5	*tcdA*, *tcdB*	*cdtAB*	CLR	23S rRNA, *blaCDD-1*, *vanZ1*, *aph(3′)-IIIa*, *ant(6)-Ia*, *tet*(40), *tet*(M), *sat-4*, *gyrA*, *gyrB*	23S rRNA: C665T
ASS24		RT126	ST11	5	*tcdA*, *tcdB*	*cdtAB*	CLR	23S rRNA, *blaCDD-1*, *vanZ1*, *aph(3′)-IIIa*, *ant(6)-Ia*, *tet*(40), *tet*(M), *sat-4*, *gyrA*, *gyrB*	23S rRNA: C665T
ASS25		RT126	ST11	5	*tcdA*, *tcdB*	*cdtAB*	CLR	23S rRNA, *blaCDD-1*, *vanZ1*, *aph(3′)-IIIa*, *ant(6)-Ia*, *tet*(40), *tet*(M), *sat-4*, *gyrA*, *gyrB*	23S rRNA: C665T
S45	S	RT014	ST2	1	*tcdA*, *tcdB*			23S rRNA, *blaCDD-1*, *vanZ1*, *vanS*, *vanG*, *vanT*, *cdeA*, *gyrA*, *gyrB*	23S rRNA: C665T
CF69	CF	RT126	ST11	5	*tcdA*, *tcdB*	*cdtAB*	CLR	23S rRNA, *blaCDD-1*, *vanZ1*, *aph(3′)-IIIa*, *ant(6)-Ia*, *tet*(40), *tet*(M), *sat-4*, *gyrA*, *gyrB*	23S rRNA: C665T
CF70		RT127	ST11	5	*tcdA*, *tcdB*	*cdtAB*		23S rRNA, *blaCDD-1*, *vanZ1*, *aph(3′)-IIIa*, *tet*(M) , *gyrA*, *gyrB*	23S rRNA: C665T
CF72		RT127	ST11	5	*tcdA*, *tcdB*	*cdtAB*		23S rRNA, *blaCDD-1*, *vanZ1*, *aph(3′)-IIIa*, *tet*(M) , *gyrA*, *gyrB*	23S rRNA: C665T
CF73		RT126	ST11	5	*tcdA*, *tcdB*	*cdtAB*	CLR	23S rRNA, *blaCDD-1*, *vanZ1*, *aph(3′)-IIIa*, *ant(6)-Ia*, *tet*(40), *tet*(M), *sat-4*, *gyrA*, *gyrB*	23S rRNA: C665T
CF74		RT126	ST11	5	*tcdA*, *tcdB*	*cdtAB*	CLR	23S rRNA, *blaCDD-1*, *vanZ1*, *aph(3′)-IIIa*, *ant(6)-Ia*, *tet*(40), *tet*(M), *sat-4*, *gyrA*, *gyrB*	23S rRNA: C665T
CF75		RT126	ST11	5	*tcdA*, *tcdB*	*cdtAB*	CLR	23S rRNA, *blaCDD-1*, *vanZ1*, *aph(3′)-IIIa*, *ant(6)-Ia*, *tet*(40), *tet*(M), *sat-4*, *gyrA*, *gyrB*	23S rRNA: C665T
CF76		RT126	ST11	5	*tcdA*, *tcdB*	*cdtAB*	CLR	23S rRNA, *blaCDD-1*, *vanZ1*, *aph(3′)-IIIa*, *ant(6)-Ia*, *tet*(40), *tet*(M), *sat-4*, *gyrA*, *gyrB*	23S rRNA: C665T
CF77		RT126	ST11	5	*tcdA*, *tcdB*	*cdtAB*	CLR	23S rRNA, *blaCDD-1*, *vanZ1*, *aph(3′)-IIIa*, *ant(6)-Ia*, *tet*(40), *tet*(M), *sat-4*, *gyrA*, *gyrB*	23S rRNA: C665T
CF78		RT126	ST11	5	*tcdA*, *tcdB*	*cdtAB*	CLR	23S rRNA, *blaCDD-1*, *vanZ1*, *aph(3′)-IIIa*, *ant(6)-Ia*, *tet*(M), *sat-4*, *gyrA*, *gyrB*	23S rRNA: C665T
CF79		RT126	ST11	5	*tcdA*, *tcdB*	*cdtAB*	CLR	23S rRNA, *blaCDD-1*, *vanZ1*, *aph(3′)-IIIa*, *ant(6)-Ia*, *tet*(40), *tet*(M), *sat-4*, *gyrA*, *gyrB*	23S rRNA: C665T
CF80		RT126	ST11	5	*tcdA*, *tcdB*	*cdtAB*	CLR, RIF	23S rRNA, *blaCDD-1*, *vanZ1*, *aph(3′)-IIIa*, *ant(6)-Ia*, *tet*(40), *tet*(M), *sat-4*, *gyrA*, *gyrB*	23S rRNA: C665T
CF81		RT126	ST11	5	*tcdA*, *tcdB*	*cdtAB*	CLR	23S rRNA, *blaCDD-1*, *vanZ1*, *aph(3′)-IIIa*, *ant(6)-Ia*, *tet*(40), *tet*(M), *sat-4*, *gyrA*, *gyrB*	23S rRNA: C665T
CF82		RT126	ST11	5	*tcdA*, *tcdB*	*cdtAB*	CLR, MXF	23S rRNA, *blaCDD-1*, *vanZ1*, *aph(3′)-IIIa*, *ant(6)-Ia*, *tet*(M), *sat-4*, *npmA*, *mefH*, *gyrA*, *gyrB*	23S rRNA: C665T
CF83		RT126	ST11	5	*tcdA*, *tcdB*	*cdtAB*		23S rRNA, *blaCDD-1*, *vanZ1*, *aph(3′)-IIIa*, *tet(M)* , *gyrA*, *gyrB*	23S rRNA: C665T
CF84		RT126	ST11	5	*tcdA*, *tcdB*	*cdtAB*	CLR, MXF	23S rRNA, *blaCDD-1*, *vanZ1*, *aph(3′)-IIIa*, *ant(6)-Ia*, *tet*(M), *sat-4*, *npmA*, *mefH*, *gyrA*, *gyrB*	23S rRNA: C665T
CF85		RT126	ST11	5	*tcdA*, *tcdB*	*cdtAB*	CLR, MXF	23S rRNA, *blaCDD-1*, *vanZ1*, *aph(3′)-IIIa*, *ant(6)-Ia*, *tet*(M), *sat-4*, *npmA*, *mefH*, *gyrA*, *gyrB*	23S rRNA: C665T
CF86		RT126	ST11	5	*tcdA*, *tcdB*	*cdtAB*	CLR, MXF	23S rRNA, *blaCDD-1*, *vanZ1*, *aph(3′)-IIIa*, *ant(6)-Ia*, *tet*(M), *sat-4*, *npmA*, *mefH*, *gyrA*, *gyrB*	23S rRNA: C665T
CF87		RT126	ST11	5	*tcdA*, *tcdB*	*cdtAB*	MXF	23S rRNA, *blaCDD-1*, *vanZ1*, *aph(3′)-IIIa*, *ant(6)-Ia*, *tet*(M), *sat-4*, *npmA*, *mefH*, *gyrA*, *gyrB*	23S rRNA: C665T
CF88		RT127	ST11	5	*tcdA*, *tcdB*	*cdtAB*		23S rRNA, *blaCDD-1*, *vanZ1*, *aph(3′)-IIIa*, *tet*(M) , *gyrA*, *gyrB*	23S rRNA: C665T
CF89		RT127	ST11	5	*tcdA*, *tcdB*	*cdtAB*		23S rRNA, *blaCDD-1*, *vanZ1*, *aph(3′)-IIIa*, *tet*(M) , *gyrA*, *gyrB*	23S rRNA: C665T
CF90		RT127	ST11	5	*tcdA*, *tcdB*	*cdtAB*		23S rRNA *blaCDD-1*, , *vanZ1*, *aph(3′)-IIIa*, *tet*(M) , *gyrA*, *gyrB*	23S rRNA: C665T
CF92		RT127	ST11	5	*tcdA*, *tcdB*	*cdtAB*		23S rRNA, *blaCDD-1*, *vanZ1*, *aph(3′)-IIIa*, *tet*(M) , *gyrA*, *gyrB*	23S rRNA: C665T
CF95		RT126	ST11	5	*tcdA*, *tcdB*	*cdtAB*	CLR, MXF	23S rRNA, *blaCDD-1*, *vanZ1*, *aph(3′)-IIIa*, *ant(6)-Ia*, *tet*(M), *sat-4*, *npmA*, *mefH*, *gyrA*, *gyrB*	23S rRNA: C665T
CF97		UC	ST11	5	*tcdA*, *tcdB*	*cdtAB*	CLR, MXF	23S rRNA, *blaCDD-1*, *vanZ1*, *aph(3′)-IIIa*, *ant(6)-Ia*, *tet*(M), *sat-4*, *npmA*, *mefH*, *gyrA*, *gyrB*	23S rRNA: C665T
CF99		RT126	ST11	5	*tcdA*, *tcdB*	*cdtAB*	CLR, MXF	23S rRNA, *blaCDD-1*, *vanZ1*, *aph(3′)-IIIa*, *ant(6)-Ia*, *tet*(M), *sat-4*, *npmA*, *mefH*, *gyrA*, *gyrB*	23S rRNA: C665T
CF101		UC	ST11	5	*tcdA*, *tcdB*	*cdtAB*		23S rRNA, *blaCDD-1*, *gyrA*, *gyrB*	23S rRNA: C665T
CF102		UC	ST11	5	*tcdA*, *tcdB*	*cdtAB*		23S rRNA, *blaCDD-1*, *gyrA*, *gyrB*	23S rRNA: C665T
CF103		UC	ST11	5	*tcdA*, *tcdB*	*cdtAB*		23S rRNA, *blaCDD-1*, *gyrA*, *gyrB*	23S rRNA: C665T
CF107		RT126	ST11	5	*tcdA*, *tcdB*	*cdtAB*		23S rRNA, *blaCDD-1*, *vanZ1*, *aph(3′)-IIIa*, *tet*(M) , *gyrA*, *gyrB*	23S rRNA: C665T
CF109		RT127	ST11	5	*tcdA*, *tcdB*	*cdtAB*		23S rRNA, *blaCDD-1*, *vanZ1*, *aph(3′)-IIIa*, *tet*(M) , *gyrA*, *gyrB*	23S rRNA: C665T
CF113		RT127	ST11	5	*tcdA*, *tcdB*	*cdtAB*		23S rRNA, *blaCDD-1*, *vanZ1*, *aph(3′)-IIIa*, *tet*(M) , *gyrA*, *gyrB*	23S rRNA: C665T
CF114		UC	ST11	5	*tcdA*, *tcdB*	*cdtAB*		23S rRNA, *blaCDD-1*, *vanZ1*, *aph(3′)-IIIa*, *tet*(M) , *gyrA*, *gyrB*	23S rRNA: C665T

Table S1: Continued.

Isolate No.	Source	RTs	STs	MLST calde	Toxin genes	Binary toxins	AR profile	AMR genes	Mutations and amino acid substitution
CF129		RT127	ST11	5	*tcdA*, *tcdB*	*cdtAB*		23S rRNA, *blaCDD-1*, *vanZ1*, *aph(3′)-IIIa*, *tet*(M) , *gyrA*, *gyrB*	23S rRNA: C665T
CF132		RT127	ST11	5	*tcdA*, *tcdB*	*cdtAB*		23S rRNA, *blaCDD-1*, *vanZ1*, *aph(3′)-IIIa*, *tet*(M) , *gyrA*, *gyrB*	23S rRNA: C665T
CF192		RT127	ST11	5	*tcdA*, *tcdB*	*cdtAB*		23S rRNA, *blaCDD-1*, 23S rRNA, *vanZ1*, *aph(3′)-IIIa*, *tet*(M) , *gyrA*, *gyrB*	23S rRNA: C665T
CF193	CF	RT127	ST11	5	*tcdA*, *tcdB*	*cdtAB*		23S rRNA, *blaCDD-1*, *vanZ1*, *aph(3′)-IIIa*, *tet*(M) , *gyrA*, *gyrB*	23S rRNA: C665T
CF194		RT127	ST11	5	*tcdA*, *tcdB*	*cdtAB*		23S rRNA, *blaCDD-1*, *vanZ1*, *aph(3′)-IIIa*, *tet*(M) , *gyrA*, *gyrB*	23S rRNA: C665T
CF195		RT127	ST11	5	*tcdA*, *tcdB*	*cdtAB*		23S rRNA, *blaCDD-1*, *vanZ1*, *aph(3′)-IIIa*, *tet*(M) , *gyrA*, *gyrB*	23S rRNA: C665T
CF196		RT127	ST11	5	*tcdA*, *tcdB*	*cdtAB*		23S rRNA, *blaCDD-1*, *vanZ1*, *aph(3′)-IIIa*, *tet*(M) , *gyrA*, *gyrB*	23S rRNA: C665T
CF200		RT126	ST11	5	*tcdA*, *tcdB*	*cdtAB*	CLR, MXF	23S rRNA, *blaCDD-1*, *vanZ1*, *aph(3′)-IIIa*, *ant(6)-Ia*, *tet*(M), *sat-4*, *npmA*, *mefH*, *gyrA*, *gyrB*	23S rRNA: C665T
BP71		RT127	ST11	5	*tcdA*, *tcdB*	*cdtAB*		23S rRNA, *blaCDD-1*, *vanZ1*, *aph(3′)-IIIa*, *tet*(M) , *gyrA*, *gyrB*	23S rRNA: C665T
BP197		RT127	ST11	5	*tcdA*, *tcdB*	*cdtAB*		23S rRNA *blaCDD-1*, , *vanZ1*, *aph(3′)-IIIa*, *tet*(M) , *gyrA*, *gyrB*	23S rRNA: C665T
BP198	BP	RT127	ST11	5	*tcdA*, *tcdB*	*cdtAB*		23S rRNA, *blaCDD-1*, *vanZ1*, *aph(3′)-IIIa*, *tet*(M) , *gyrA*, *gyrB*	23S rRNA: C665T
BP199		RT127	ST11	5	*tcdA*, *tcdB*	*cdtAB*		23S rRNA, *blaCDD-1*, *vanZ1*, *aph(3′)-IIIa*, *tet*(M) , *gyrA*, *gyrB*	23S rRNA: C665T
BP201		RT127	ST11	5	*tcdA*, *tcdB*	*cdtAB*		23S rRNA, *blaCDD-1*, *vanZ1*, *aph(3′)-IIIa*, *tet*(M) , *gyrA*, *gyrB*	23S rRNA: C665T
TDS115		RT001	ST3	1	*tcdA*, *tcdB*			23S rRNA, *blaCDD-1*, *vanZ1*, *vanS*, *vanG*, *vanT*, *gyrA*, *gyrB*	23S rRNA: C665T
TDS116		RT076	ST2	1	*tcdA*, *tcdB*			23S rRNA, *blaCDD-1*, *vanS*, *vanG*, *vanT*, *gyrA*, *gyrB*	23S rRNA: C665T
TDS117		RT014	ST49	1	*tcdA*, *tcdB*			23S rRNA, *blaCDD-1*, *vanS*, *vanG*, *vanT*, *gyrA*, *gyrB*	23S rRNA: C665T
TDS118		RT140	ST515	1			CLR, MXF	23S rRNA, *blaCDD-1*, *vanZ1*, *vanS*, *vanG*, *vanT*, *aph(2′′)-If*, *tet*(M), *tet*(O), *ermB*, *ant(6)-Ia*, *gyrA*	23S rRNA: C665T *gyrA*: T82I
TDS119	TDS	RT014	ST2	1	*tcdA*, *tcdB*			23S rRNA, *blaCDD-1*, *vanS*, *vanG*, *vanT*, *gyrA*, *gyrB*	23S rRNA: C665T
TDS120		RT076	ST2	1	*tcdA*, *tcdB*			23S rRNA, *blaCDD-1*, *vanS*, *vanG*, *vanT*, *gyrA*, *gyrB*	23S rRNA: C665T
TDS121		RT076	ST2	1	*tcdA*, *tcdB*			23S rRNA, *blaCDD-1*, *vanS*, *vanG*, *vanT*, *gyrA*, *gyrB*	23S rRNA: C665T
TDS122		RT076	ST2	1	*tcdA*, *tcdB*			23S rRNA, *blaCDD-1*, *vanS*, *vanG*, *vanT*, *gyrA*, *gyrB*	23S rRNA: C665T
TDS128		RT127	ST11	5	*tcdA*, *tcdB*	*cdtAB*		23S rRNA, *blaCDD-1*, *vanZ1*, *aph(3′)-IIIa*, *tet*(M) , *gyrA*, *gyrB*	23S rRNA: C665T
TDB123		RT001	ST3	1	*tcdA*, *tcdB*			23S rRNA, *blaCDD-1*, *vanZ1*, *vanS*, *vanG*, *vanT*, *gyrA*, *gyrB*	23S rRNA: C665T
TDB124		RT023	ST5	3	*tcdA*, *tcdB*	*cdtAB*		23S rRNA, *blaCDD-1*, *vanZ1*, *gyrA*, *gyrB*	23S rRNA: C665T
TDB126	TDB	RT001	ST3	1	*tcdA*, *tcdB*			23S rRNA, *blaCDD-1*, *vanZ1*, *vanS*, *vanG*, *vanT*, *gyrA*, *gyrB*	23S rRNA: C665T
TDB127		RT023	ST5	3	*tcdA*, *tcdB*	*cdtAB*		23S rRNA, *blaCDD-1*, *vanZ1*, *gyrA*, *gyrB*	23S rRNA: C665T
TDB130		RT001	ST3	1	*tcdA*, *tcdB*			23S rRNA, *blaCDD-1*, *vanZ1*, *vanS*, *vanG*, *vanT*, *gyrA*, *gyrB*	23S rRNA: C665T
TDB131		RT001	ST3	1	*tcdA*, *tcdB*			23S rRNA, *blaCDD-1*, *vanZ1*, *vanS*, *vanG*, *vanT*, *gyrA*, *gyrB*	23S rRNA: C665T
ARC134		RT095	ST2	1	*tcdA*, *tcdB*			23S rRNA, *blaCDD-1*, *vanS*, *vanG*, *vanT*, *gyrA*, *gyrB*	23S rRNA: C665T
ARC135		RT077	ST13	1	*tcdA*, *tcdB*			23S rRNA, *blaCDD-1*, *vanZ1*, *vanS*, *vanG*, *vanT*, *gyrA*, *gyrB*	23S rRNA: C665T
ARC139	ARC	RT015	ST44	1	*tcdA*, *tcdB*		MXF	23S rRNA, *blaCDD-1*, *vanZ1*, *vanS*, *vanG*, *vanT*, *gyrA*, *gyrB*	23S rRNA: C665T *gyrA*: D71G
ARC140		RT014	ST2	1	*tcdA*, *tcdB*		CLR	23S rRNA, *blaCDD-1*, *vanZ1*, *vanS*, *vanG*, *vanT*, *aac(6′)-aph(2′′)*, *ermB*, *gyrA*, *gyrB*	23S rRNA: C665T
ARC141		RT020	ST2	1	*tcdA*, *tcdB*			23S rRNA, *blaCDD-1*, *vanS*, *vanG*, *vanT*, *gyrA*, *gyrB*	23S rRNA: C665T

Table S1: Continued.

Isolate No.	Source	RTs	STs	MLST calde	Toxin genes	Binary toxins	AR profile	AMR genes	Mutations and amino acid substitution
ARC167		UC	ST8	1	*tcdA*, *tcdB*			23S rRNA, *blaCDD-1*, *vanZ1*, *vanS*, *vanG*, *vanT*, *gyrA*, *gyrB*	23S rRNA: C665T
ARC168	ARC	RT159	ST8	1	*tcdA*, *tcdB*			23S rRNA, *blaCDD-1*, *vanZ1*, *vanS*, *vanG*, *vanT*, *gyrA*, *gyrB*	23S rRNA: C665T
ARC182		RT005	ST6	1	*tcdA*, *tcdB*			23S rRNA, *blaCDD-1*, *vanZ1*, *vanS*, *vanG*, *vanT*, *gyrA*, *gyrB*	23S rRNA: C665T
ARE136		RT120	ST4	1	*tcdA*, *tcdB*			23S rRNA, *blaCDD-2*, *vanZ1*, *vanS*, *vanG*, *vanT*, *gyrA*, *gyrB*	23S rRNA: C665T
ARE137		RT120	ST4	1	*tcdA*, *tcdB*			23S rRNA, *blaCDD-2*, *vanZ1*, *vanS*, *vanG*, *vanT*, *gyrA*, *gyrB*	23S rRNA: C665T
ARE138		RT120	ST4	1	*tcdA*, *tcdB*			23S rRNA, *blaCDD-2*, *vanZ1*, *vanS*, *vanG*, *vanT*, *gyrA*, *gyrB*	23S rRNA: C665T
ARE143		RT120	ST4	1	*tcdA*, *tcdB*			23S rRNA, *blaCDD-2*, *vanZ1*, *vanS*, *vanG*, *vanT*, *gyrA*, *gyrB*	23S rRNA: C665T
ARE144	ARE	RT120	ST4	1	*tcdA*, *tcdB*			23S rRNA, *blaCDD-2*, *vanZ1*, *vanS*, *vanG*, *vanT*, *gyrA*, *gyrB*	23S rRNA: C665T
ARE145		UC	ST109	4				23S rRNA, *blaCDD-2*, *vanZ1*, *gyrA*, *gyrB*	23S rRNA: C665T
ARE146		RT085	ST39	4	*tcdA*, *tcdB*		CLR, RIF	23S rRNA, *blaCDD-2*, *vanZ1*, *ermB*, *tet*(M), *rpoB*, *gyrA*, *gyrB*	23S rRNA: C665T<br>*rpoB*: His502Asn/Arg505Lys
ARE170		RT120	ST4	1	*tcdA*, *tcdB*			23S rRNA, *blaCDD-2*, *vanZ1*, *vanS*, *vanG*, *vanT*, *gyrA*, *gyrB*	23S rRNA: C665T
DS155		RT078	ST11	5	*tcdA*, *tcdB*	*cdtAB*		23S rRNA, *blaCDD-1*, *vanZ1*, *ermB*, *gyrA*, *gyrB*	23S rRNA: C665T
DS156		RT078	ST11	5	*tcdA*, *tcdB*	*cdtAB*	CLR	23S rRNA, *blaCDD-1*, *vanZ1*, *ermB*, *gyrA*, *gyrB*	23S rRNA: C665T
DS157		RT001	ST3	1	*tcdA*, *tcdB*		CLR, MXF	23S rRNA, *blaCDD-1*, *vanS*, *vanG*, *vanT*, *aac(6′)-aph(2′′)*, *tetB*(P), *tetA*(P), *ermB*, *gyrA*, *gyrB*	23S rRNA: C665T<br>*gyrA*: T82I
DS158		RT126	ST11	5	*tcdA*, *tcdB*	*cdtAB*	CLR, MXF	23S rRNA, *blaCDD-1*, *vanZ1*, *aph(3′)-IIIa*, *ant(6)-Ia*, *tet*(M), *sat-4*, *ermB*, *gyrA*, *gyrB*, *aad9*	23S rRNA: C665T<br>*gyrA*: T82A
DS159		RT126	ST11	5	*tcdA*, *tcdB*	*cdtAB*	CLR, MXF	23S rRNA, *blaCDD-1*, *vanZ1*, *aph(3′)-IIIa*, *ant(6)-Ia*, *tet*(M), *sat-4*, *ermB*, *gyrA*, *gyrB*, *aad9*, *tet*(40)	23S rRNA: C665T<br>*gyrA*: T82A
DS160		UC	ST1074	UC				23S rRNA, *blaCDD-2*	23S rRNA: C665T
DS161		RT001	ST3	1	*tcdA*, *tcdB*		CLR, MXF	23S rRNA, *blaCDD-1*, *vanS*, *vanG*, *vanT*, *aac(6′)-aph(2′′)*, *tetB*(P), *tetA*(P), *ermB*, *gyrA*, *gyrB*	23S rRNA: C665T<br>*gyrA*: T82I
DS162	DSS-S	RT078	ST11	5	*tcdA*, *tcdB*	*cdtAB*	MXF	23S rRNA, *blaCDD-1*, *vanZ1*, *aph(3′)-IIIa*, *sat-4*, *ant(6)-la*, *gyrA*, *gyrB*	23S rRNA: C665T<br>*gyrA*: T82I
DS163		RT001	ST3	1	*tcdA*, *tcdB*			23S rRNA, *blaCDD-1*, *vanZ1*, *vanS*, *vanG*, *vanT*, *gyrA*, *gyrB*	23S rRNA: C665T
DS169		UC	ST821	UC				23S rRNA, *blaCDD-2*, *gyrA*, *gyrB*	23S rRNA: C665T
DS171		RT078	ST11	5	*tcdA*, *tcdB*	*cdtAB*	MXF	23S rRNA, *blaCDD-1*, *vanZ1*, *aph(3′)-IIIa*, *ant(6)-Ia*, *tet*(M), *sat-4*, *gyrA*, *gyrB*, *spw*	23S rRNA: C665T<br>*gyrA*: T82I
DS173		RT078	ST11	5	*tcdA*, *tcdB*	*cdtAB*	MXF	23S rRNA, *blaCDD-1*, *vanZ1*, *aph(3′)-IIIa*, *ant(6)-Ia*, *sat-4*, *gyrA*, *gyrB*	23S rRNA: C665T<br>*gyrA*: T82I
DS174		RT078	ST11	5	*tcdA*, *tcdB*	*cdtAB*	MXF	23S rRNA, *blaCDD-1*, *vanZ1*, *aph(3′)-IIIa*, *ant(6)-Ia*, *sat-4*, *gyrA*, *gyrB*	23S rRNA: C665T<br>*gyrA*: T82I
DS175		RT078	ST11	5	*tcdA*, *tcdB*	*cdtAB*	CLR	23S rRNA, *blaCDD-1*, *vanZ1*, *sat-4*, *ant(6)-Ia*, *tet*(40), *aph(3′)-IIIa*, *gyrA*, *gyrB*	23S rRNA: C665T
DS176		RT078	ST11	5	*tcdA*, *tcdB*	*cdtAB*	CLR	23S rRNA, *blaCDD-1*, *vanZ1*, *sat-4*, *ant(6)-Ia*, *tet*(40), *aph(3′)-IIIa*	23S rRNA: C665T

Table S1: Continued.

Isolate No.	Source	RTs	STs	MLST calde	Toxin genes	Binary toxins	AR profile	AMR genes	Mutations and amino acid substitution
DS177	DSS-S	RT078	ST11	5	*tcdA*, *tcdB*	*cdtAB*	MXF, CLR	23S rRNA, *blaCDD-1*, *vanZ1*, *ermB*, *gyrA*, *gyrB*	23S rRNA: C665T
DS178		RT120	ST4	1	*tcdA*, *tcdB*			23S rRNA, *blaCDD-2*, *vanZ1*, *vanS*, *vanG*, *vanT*, *gyrA*, *gyrB*	23S rRNA: C665T
DS179		RT078	ST11	5	*tcdA*, *tcdB*	*cdtAB*		23S rRNA, *blaCDD-1*, *vanZ1*, *spw*, *ant(6)-Ia*, *tet*(M), *gyrA*, *gyrB*	23S rRNA: C665T
DS180		RT078	ST11	5	*tcdA*, *tcdB*	*cdtAB*		23S rRNA, *blaCDD-1*, *vanZ1*, *spw*, *ant(6)-Ia*, *tet*(M) , *gyrA*, *gyrB*	23S rRNA: C665T
DS181		RT126	ST11	5	*tcdA*, *tcdB*	*cdtAB*	MXF, CLR	23S rRNA, *blaCDD-1*, *vanZ1*, *aph(3')-IIIa*, *ant(6)-Ia*, *tet*(M), *sat-4*, *aad9*, *gyrA*, *gyrB*, *tet*(40), *ermB*	23S rRNA: C665T<br>*gyrA*: T82A

RSS: raw sewage sludge, AR: antimicrobial resistance, AMR: antimicrobial resistance, CLR: clarithromycin, MXF: moxifloxacin, RIF: rifampicin, RS: raw sewage, ASS: activated sewage sludge, DSS: digested sewage sludge, CF: calf feces, BP: biogas plant digestate, S: soil; ARC/E: anaerobic lab scale bioreactors treating sewage sludge (control and experiment), DSS-S: digested sewage sludge-amended soils, TDS: thermophilic digester for treating sewage sludge, TDB: thermophilic digester for treating biowaste, UC: unclassified.

Table S2: Distribution of *C. difficile* RTs (*n* = 166) in diverse Environmental samples.

Source	RTs															
	005	090	011	014	070	159	015	012	010	140	023	018	001	UC	078	020
ASS	0	0	0	2	0	0	0	1	0	0	0	0	0	0	0	0
RS	0	0	1	1	2	0	1	1	0	0	1	1	4	1	0	0
RSS	1	1	1	0	1	1	0	1	1	2	1	0	1	1	0	1
DSS	0	0	0	1	0	0	1	0	0	0	0	2	0	3	0	2
CF	0	0	0	0	0	0	0	0	0	0	0	0	0	5	0	0
BP	0	0	0	0	0	0	0	0	0	0	0	0	0	0	0	0
TD/TDB	0	0	0	2	0	0	0	0	0	1	2	0	5	0	0	0
DSS-S	0	0	0	0	0	0	0	0	0	0	0	0	3	2	11	0
S	0	0	0	1	0	0	0	0	0	0	0	0	0	0	0	0
ARC/E	1	0	0	1	0	1	1	0	0	0	0	0	0	2	0	1
**Total**	2	1	2	8	3	2	3	3	1	3	4	3	13	14	11	4

Source	RTs														
	126	258	106	103	031	017	002	127	076	095	077	120	085	073	328
ASS	3	0	0	0	0	0	0	0	0	0	0	0	0	0	0
RS	0	0	0	0	0	0	1	0	0	0	0	0	0	7	0
RSS	0	0	0	0	1	1	0	8	0	0	0	0	0	0	0
DSS	1	3	2	1	0	0	1	0	0	0	0	0	0	0	2
CF	20	0	0	0	0	0	0	15	0	0	0	0	0	0	0
BP	0	0	0	0	0	0	0	5	0	0	0	0	0	0	0
TD/TDB	0	0	0	0	0	0	0	1	4	0	0	0	0	0	0
DSS-S	3	0	0	0	0	0	0	0	0	0	0	1	0	0	0
S	0	0	0	0	0	0	0	0	0	0	0	0	0	0	0
ARC/E	0	0	0	0	0	0	0	0	0	1	1	6	1	0	0
**Total**	27	3	2	1	1	1	2	29	4	1	1	7	1	7	2

ASS: activated sewage sludge, RS: raw sewage, RSS: raw sewage sludge, DSS: digested sewage sludge, CF: calf feces, BP: biogas plant digestate, TD/TDB: thermophilic digester for sewage sludge or biowaste, DSS-S: digested sludge-amended soils, S: soil; ARC/E: anaerobic lab scale bioreactors treating sewage sludge (control and experiment), UC: unclassified.

Table S3: Toxin-encoding gene profiles of *C. difficile* RT strains (*n* = 166) from various environmental samples. UC: unclassified.

RT	No. of strains	*tcd*A^{+}/*tcdB*$^{+}$	*tcdA*$^{+}$/*tcdB*$^{+}$/*cdtAB*$^{+}$	*tcd*A^{-}/*tcdB*$^{-}$/ *cdtAB*$^{-}$
RT005	2	2	0	0
RT090	1	1	0	0
RT011	2	2	0	0
RT020	4	4	0	0
RT014	8	8	0	0
RT070	3	3	0	0
RT159	2	2	0	0
RT012	3	3	0	0
RT010	1	0	0	1
RT140	3	0	0	3
RT023	4	0	4	0
RT031	1	0	0	1
RT001	13	13	0	0
RT017	1	1	0	0
RT127	29	0	29	0
RT015	3	3	0	0
RT018	3	3	0	0
RT002	2	2	0	0
RT073	7	0	0	7
RT258	3	3	0	0
RT126	27	0	27	0
RT076	4	4	0	0
RT095	1	1	0	0
RT077	1	1	0	0
RT120	7	7	0	0
RT085	1	1	0	0
RT078	11	0	11	0
RT106	2	2	0	0
RT328	2	2	0	0
RT103	1	1	0	0
UC	15	3	5	6
Total	166	72	76	18

Table S4: Distribution of *C. difficile* STs (*n* = 166) in diverse environmental samples.

Source	STs																														
	821	6	1073	36	2	55	8	44	254	54	15	26	5	14	17	39	1074	3	917	11	58	42	29	37	13	325	49	515	4	109	35	53
ASS	0	0	0	0	2	0	0	0	0	1	0	0	0	0	0	0	0	0	0	3	0	0	0	0	0	0	0	0	0	0	0	0
RS	0	0	0	0	0	2	1	1	1	1	0	0	1	1	1	0	0	4	0	0	0	0	0	0	0	1	0	0	0	7	0	0
RSS	0	2	1	1	1	1	1	0	0	1	1	2	1	0	0	0	0	1	0	8	0	0	1	1	0	0	0	0	0	0	0	0
DSS	0	0	0	0	2	0	2	1	1	0	0	0	0	0	2	0	0	0	1	1	3	2	0	0	1	0	0	0	0	0	2	1
CF	0	0	0	0	0	0	0	0	0	0	0	0	0	0	0	0	0	0	0	40	0	0	0	0	0	0	0	0	0	0	0	0
BP	0	0	0	0	0	0	0	0	0	0	0	0	0	0	0	0	0	0	0	5	0	0	0	0	0	0	0	0	0	0	0	0
TD/TDB	0	0	0	0	5	0	0	0	0	0	0	0	2	0	0	0	0	5	0	1	0	0	0	0	0	0	1	1	0	0	0	0
DSS-S	1	0	0	0	0	0	0	0	0	0	0	0	0	0	0	0	1	3	0	14	0	0	0	0	0	0	0	0	0	0	0	0
S	0	0	0	0	1	0	0	0	0	0	0	0	0	0	0	0	0	0	0	0	0	0	0	0	0	0	0	0	0	0	0	0
AR-C/-E	0	1	0	0	3	0	2	1	0	0	0	0	0	0	0	1	0	0	0	0	0	0	0	0	1	0	0	0	6	1	0	0
Total	1	3	1	1	13	3	6	3	2	3	1	2	4	1	3	1	1	13	1	72	3	2	1	1	2	1	1	1	6	8	2	1

ASS: activated sewage sludge, RS: raw sewage, RSS: raw sewage sludge, DSS: digested sewage sludge, CF: calf feces, BP: biogas plants, TD/TDB: thermophilic digester for sewage sludge or biowaste, DSS-S: digested sludge-amended soils, S: soil; ARC/E: anaerobic lab scale bioreactors treating sewage sludge (control and experiment).

Table S5: Toxin-encoding gene profiles of *C. difficile* ST strains (n = 166) isolated from various environmental samples.

ST	No. of strains	*tcd*A+/*tcdB*+	*tcdA*+/*tcdB*+/*cdtAB*+	*tcd*A-/*tcdB*-/ *cdtAB* -
ST6	3	3	0	0
ST1073	1	1	0	0
ST36	1	1	0	0
ST2	14	14	0	0
ST55	3	3	0	0
ST8	6	6	0	0
ST54	3	3	0	0
ST15	1	0	0	1
ST26	2	0	0	2
ST5	4	0	4	0
ST29	1	0	0	1
ST3	13	13	0	0
ST37	1	1	0	0
ST11	72	0	72	0
ST44	3	3	0	0
ST254	2	0	0	2
ST14	1	1	0	0
ST17	3	3	0	0
ST325	1	1	0	0
ST109	8	0	0	8
ST917	1	0	0	1
ST58	3	3	0	0
ST13	2	2	0	0
ST35	2	2	0	0
ST53	1	1	0	0
ST42	2	2	0	0
ST515	1	0	0	1
ST4	7	7	0	0
ST39	1	1	0	0
ST1074	1	0	0	1
ST821	1	0	0	1
ST49	1	1	0	0
Total	166	72	76	18

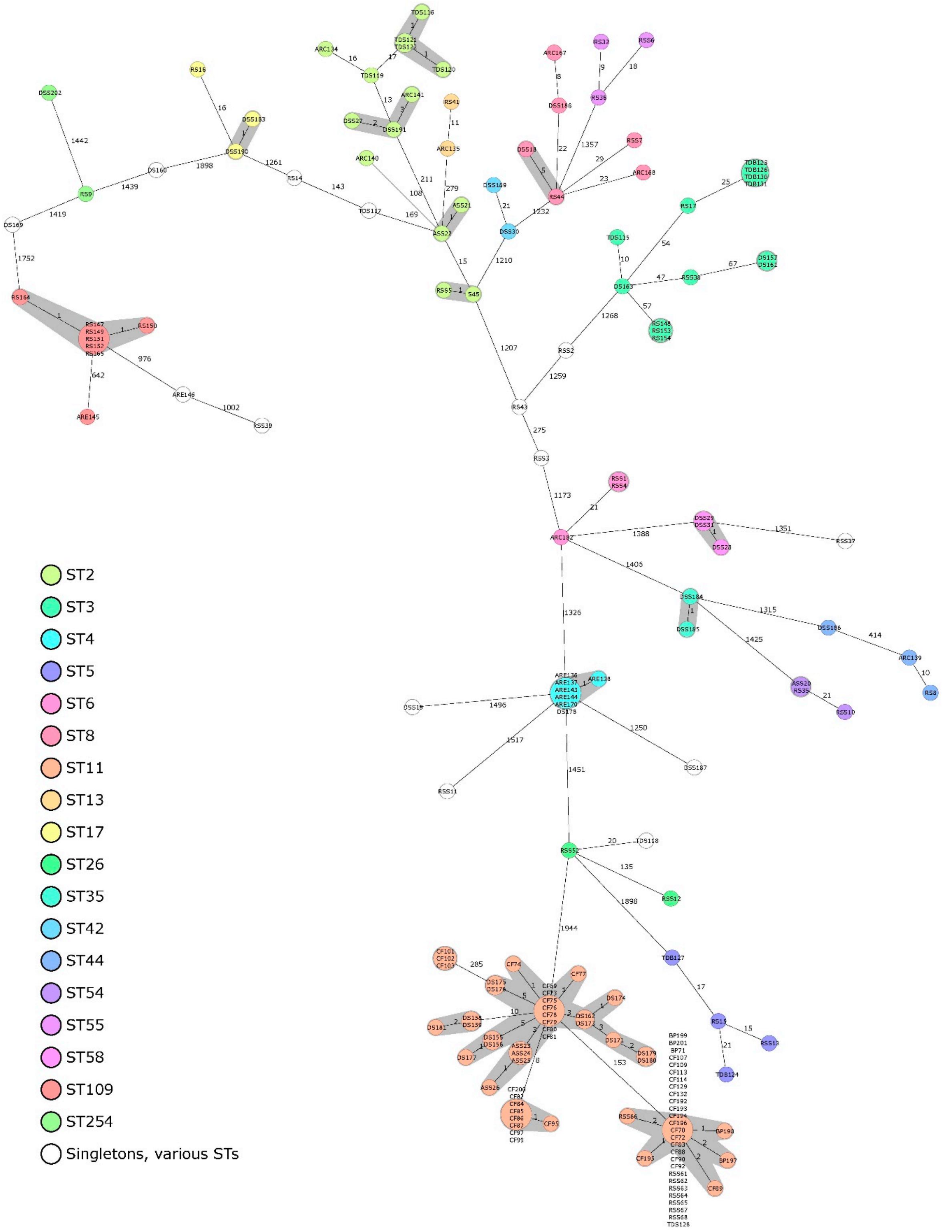

Figure S1: Minimum-spanning tree based on allelic profiles of 166 *C. difficile* isolates. Each circle represents a separate genotype and distances between two genotypes are based on the allelic profiles of up to 2,147 target genes, pairwise ignoring missing targets. The values on the connecting lines indicate the number of allelic differences between the connected isolates. Circle sizes are proportional to the numbers of isolates per genotype (i.e., the allelic profile). Related genotypes (≤6 alleles distance) are shaded in grey and the isolates are colored according to their ST. RSS: raw sewage sludge, RS: raw sewage, ASS: activated sewage sludge, DSS: digested sewage sludge, CF: calf feces, BP: biogas plant digestate, ARC/E: anaerobic lab-scale bioreactors treating sewage sludge (control and experiment), DSS-S: digested sewage sludge-amended soils, TDS: thermophilic digester for treating sewage sludge, TDB: thermophilic digester for treating biowaste, S: soil.
